# Herpes simplex viruses activate phospholipid scramblase to redistribute phosphatidylserines and Akt to the outer leaflet of the plasma membrane and promote viral entry

**DOI:** 10.1371/journal.ppat.1006766

**Published:** 2018-01-02

**Authors:** Natalia Cheshenko, Carl Pierce, Betsy C. Herold

**Affiliations:** 1 Department of Pediatrics, Albert Einstein College of Medicine, Bronx, New York, United States of America; 2 Department of Microbiology-Immunology, Albert Einstein College of Medicine, Bronx, New York, United States of America; Louisiana State University Health Sciences Center, UNITED STATES

## Abstract

Herpes simplex virus (HSV) entry is associated with Akt translocation to the outer leaflet of the plasma membrane to promote a complex signaling cascade. We hypothesized that phospholipid scramblase-1 (PLSCR1), a calcium responsive enzyme that flips phosphatidylserines between membrane leaflets, might redistribute Akt to the outside during entry. Confocal imaging, biotinylation of membrane proteins and flow cytometric analysis demonstrated that HSV activates PLSCR1 and flips phosphatidylserines and Akt to the outside shortly following HSV-1 or HSV-2 exposure. Translocation was blocked by addition of a cell permeable calcium chelator, pharmacological scramblase antagonist, or transfection with small interfering RNA targeting PLSCR1. Co-immunoprecipitation and proximity ligation studies demonstrated that PLSCR1 associated with glycoprotein L at the outer leaflet and studies with gL deletion viruses indicate that this interaction facilitates subsequent restoration of the plasma membrane architecture. Ionomycin, a calcium ionophore, also induced PLSCR1 activation resulting in Akt externalization, suggesting a previously unrecognized biological phenomenon.

## Author summary

Defining the mechanisms by which herpes simplex viruses (HSV) enter cells will facilitate the development of new strategies to prevent or treat infections and provide insights into cell biology. We report the novel observation that HSV activates the enzyme, scramblase, which redistributes phosphatidylserines, the major component of the inner leaflet of the plasma membrane, and the associated protein, Akt, between the inner and outer leaflet of the plasma membrane, to promote viral entry.

## Introduction

Herpes simplex virus serotypes 1 and 2 (HSV-1 and HSV-2) are significant global health problems, disproportionately impacting developing countries and fueling the HIV epidemic. HSV-2 is the leading cause of genital ulcerative disease worldwide, whereas HSV-1 has emerged as the more common cause of genital infection in industrialized nations [1]. Perinatal transmission of either serotype can result in severe infant morbidity or death. Moreover, HSV-1 is the most common cause of sporadic fatal encephalitis and even with optimal intravenous acyclovir therapy, mortality is 14–19% and fewer than 50% of the survivors resume a normal lifestyle [2]. These epidemiological findings highlight the need to better define the mechanisms by which HSV invades cells to establish life-long persistent infection and to exploit this knowledge to develop new antiviral strategies.

HSV entry is initiated by attachment of HSV-1 glycoprotein (g) C (gC-1) or HSV-2 gB (gB-2) to heparan sulfate moieties on syndecan proteoglycans [3–7], followed by engagement of one of several gD receptors, most commonly nectin-1 on epithelial cells [8–11]. This activates a complex signaling cascade that requires interactions between cellular molecules and viral glycoproteins gB, gH and gL and culminates in the insertion of the gB fusion loops into the plasma membrane (or less commonly, endosomal membrane), with formation of a fusion pore through which the viral capsid and tegument proteins are delivered [12,13].

Precisely how these viral glycoproteins interact with cellular components to promote viral entry is not fully defined. In previous work, we found that chelation or pharmacological blockade of intracellular Ca^2+^ release prevented HSV entry in multiple cell types [4,14]. A small amount of Ca^2+^ was detected near the plasma membrane in response to heparan sulfate binding and nectin engagement by Ca^2+^ fluorimetry and confocal microscopy. This initial Ca^2+^ response facilitated subsequent activation of Akt and downstream signaling pathways culminating in the release of inositol-triphosphate receptor (IP3R)-regulated intracellular Ca^2+^ stores that promote viral entry [4,15–17]. Co-immunoprecipitation and proximity ligation studies demonstrated that Akt associated with gB, which was surprising as Akt is presumed to shuttle between the cytoplasm and the inner leaflet of the plasma membrane, whereas viral envelope glycoproteins are retained at the outer surface. We subsequently showed that HSV triggered the externalization of Akt, providing access to the viral envelope. Akt was detected by fluorescently conjugated antibodies (Abs) in non-permeabilized cells shortly after exposure to HSV-1 or HSV-2 by confocal imaging, but was only visualized after permeabilization in mock-infected cells [16,17].

There are several transmembrane lipid transporter enzymes that translocate phospholipids between the inner (dominated by negatively charged phosphatidylserines (PtdS) and outer (dominated by phosphatidylcholine and sphingomyelin) lipid bilayer of cell membranes. These include phospholipid flippases and floppases, which are ATP-dependent unidirectional enzymes that move lipids from the outer to the cytosolic face or in the reverse direction, respectively, and phospholipid scramblases (PLSCRs), which are Ca^2+^ sensitive small transmembrane proteins that act bidirectionally. There are several splice variants of PLSCR, but PLSCR1 is the prototype and the most studied member of the family [18]. More recently, two additional scramblase proteins, TMEM16F, which is regulated by elevated intracellular Ca^2+^, and XKR8, a caspase-sensitive protein required for PtdS exposure in apoptotic cells, have been identified [19]. Mutations in TMEM16F are associated with Scott syndrome, a platelet disorder in which Ca^2+^-induced PtdS exposure is impaired [20].

We hypothesized that the initial intracellular Ca^2+^ response to HSV receptor engagement activates Ca^2+^ sensitive scramblases to trigger the translocation of PtdS from the inner to the outer leaflet of the plasma membrane resulting also in the externalization of Akt. However, because PtdS on the outer leaflet of the plasma membrane is associated with activation of apoptotic pathways [19,21], we further hypothesized that HSV would flip PtdS and Akt back to restore the plasma membrane architecture. Focusing on PLSCR1, we used a combination of confocal imaging, molecular and biochemical techniques and wild type or deletion viruses to test these hypotheses in human cervical or vaginal epithelial cells and keratinocytes. To address whether the response is limited to HSV or a more generalized phenomenon, we also examined the response to the Ca^2+^ ionophore, ionomycin.

## Results

### HSV redistributes phosphatidylserines to the outer leaflet of the plasma membrane

**Fig 1:**
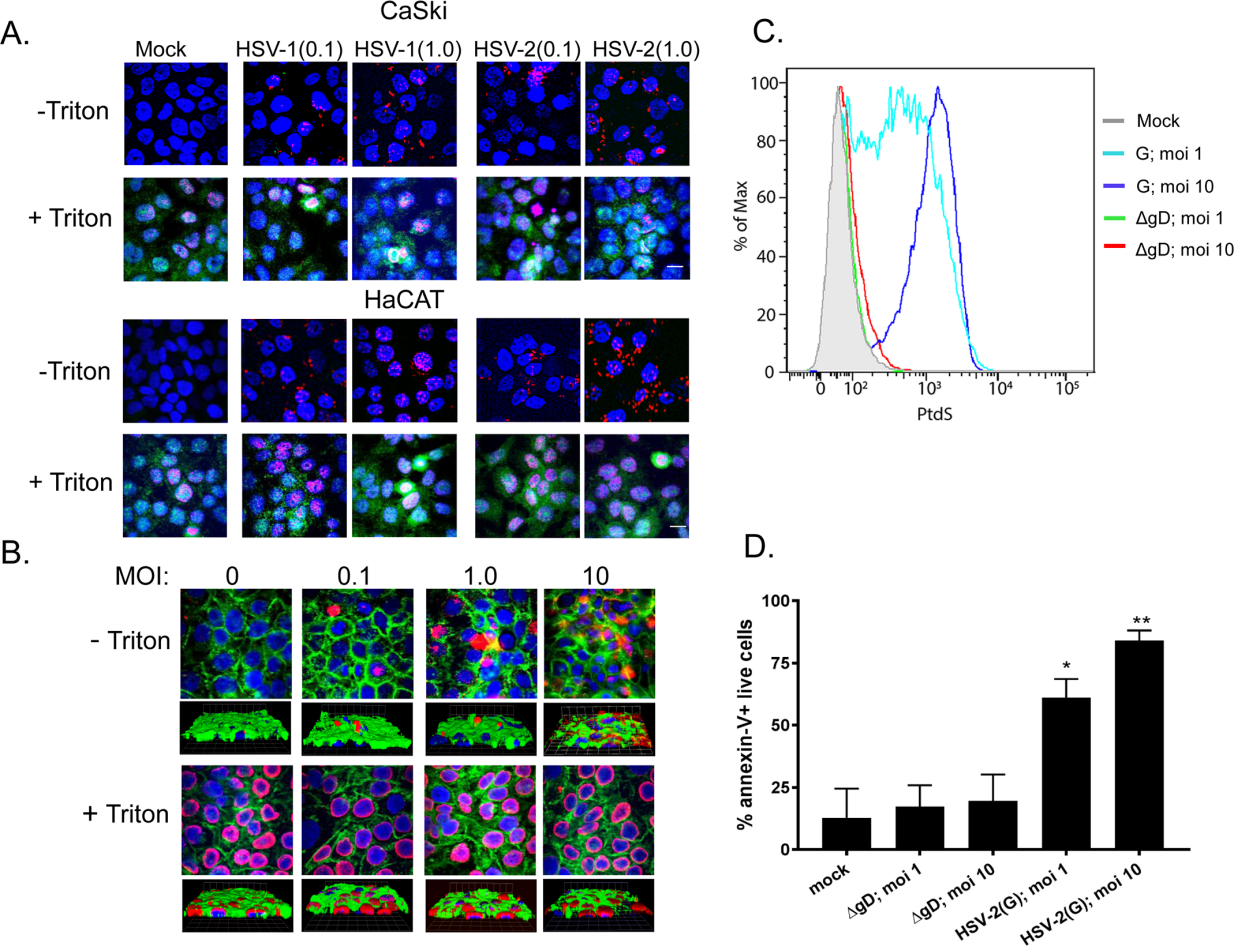
HSV triggers externalization of phosphatidylserine. (A). CaSki or HaCAT cells were synchronously infected with purified HSV-1(KOS) or HSV-2(G) at MOI of 0.1 or 1 pfu/cell (1 hour at 4°C, washed, then shifted to 37°C for 15 minutes and treated with low pH citrate buffer before being fixed with or without 0.1%Triton X). Nuclei were stained blue with DAPI, phosphatidylserines red with annexin-V conjugated with Alexa555 and flippase (FIC-1) green with rabbit polyclonal anti-flipppase (FIC-1) and secondary anti-rabbit Alexa 488. Images are representative of results obtained from 2 independent experiments; bar = 10μm. (B). HaCAT cells were prestained green with Alexa Fluor 488-conjugated wheat germ agglutinin and then exposed at 37°C to increasing MOI (0–10 pfu/cell) of HSV-2(G) and fixed and stained 30 min pi. Nuclei were stained blue with Hoechst and phosphatidylserines red with a primary murine and secondary Alexa Fluor 555-conjugated secondary antibody. Images are representative of results obtained from 2 independent experiments. Bar = 10μm. (C and D) HaCAT cells were mock-infected or infected with purified HSV-2(G) (MOI 1 or 10 pfu/cell based on Vero cell viral titer) or non-complemented HSV-2ΔgD^-/-^ (relatively equivalent viral particles based on Western blots for VP5) for 20 minutes and then live cells stained for phosphatidylserines using PE-annexin-V. A representative histogram is shown (C) and results from 4 independent experiments are presented as the % annexin-V-positive live cells (mean + SEM) (D). The asterisks indicate significance compared to mock-infected cells by ANOVA; * p< 0.05 and ** p< 0.01).

To test the hypothesis that PtdS translocate from the inner to the outer leaflet of the plasma membrane in response to HSV, human cervical epithelial cells (CaSki) or keratinocytes (HaCAT) were infected with HSV-1(KOS) or HSV-2(G) at a multiplicity of infection (MOI) of 0.1 or 1 pfu/cell at 4°C for 1 h, unbound virus removed by washing the cells and then shifted to 37°C for 15 minutes, washed with a pH 3.5 buffer to inactivate any non-penetrated virus, fixed with or without Triton X and stained with annexin V, which binds PtdS (red) or, as a control, with an antibody (Ab) to the ATP-dependent flippase, aminophospholipid transporter class 1 (FIC-1) (green). Nuclei were stained with DAPI (blue). PtdS were detected in non-permeabilized cells following exposure to HSV-1 or HSV-2 in both cell types, but FIC-1 was only detected after permeabilization (**Fig 1A**). Similar results were obtained when cells (membranes stained green and nuclei blue) were exposed to increasing concentrations of HSV-2(G) at 37°C and stained with a primary anti-PtdS monoclonal and secondary Alexa Fluor conjugated Abs (red) (**Fig 1B**). Moreover, flow cytometry also demonstrated that PtdS were detected in a significantly higher percentage of non-permeabilized HaCAT cells 20 minutes post-exposure to HSV-2(G), but not following exposure to an HSV-2(G) virus deleted in gD (HSV-2ΔgD^-/-^) (Fig 1C and 1D). The latter binds to cells, but does not engage nectin-1 or elicit any Ca^2+^ response [4,17].

### PLSCR1 is required for PtdS and Akt externalization

**Fig 2:**
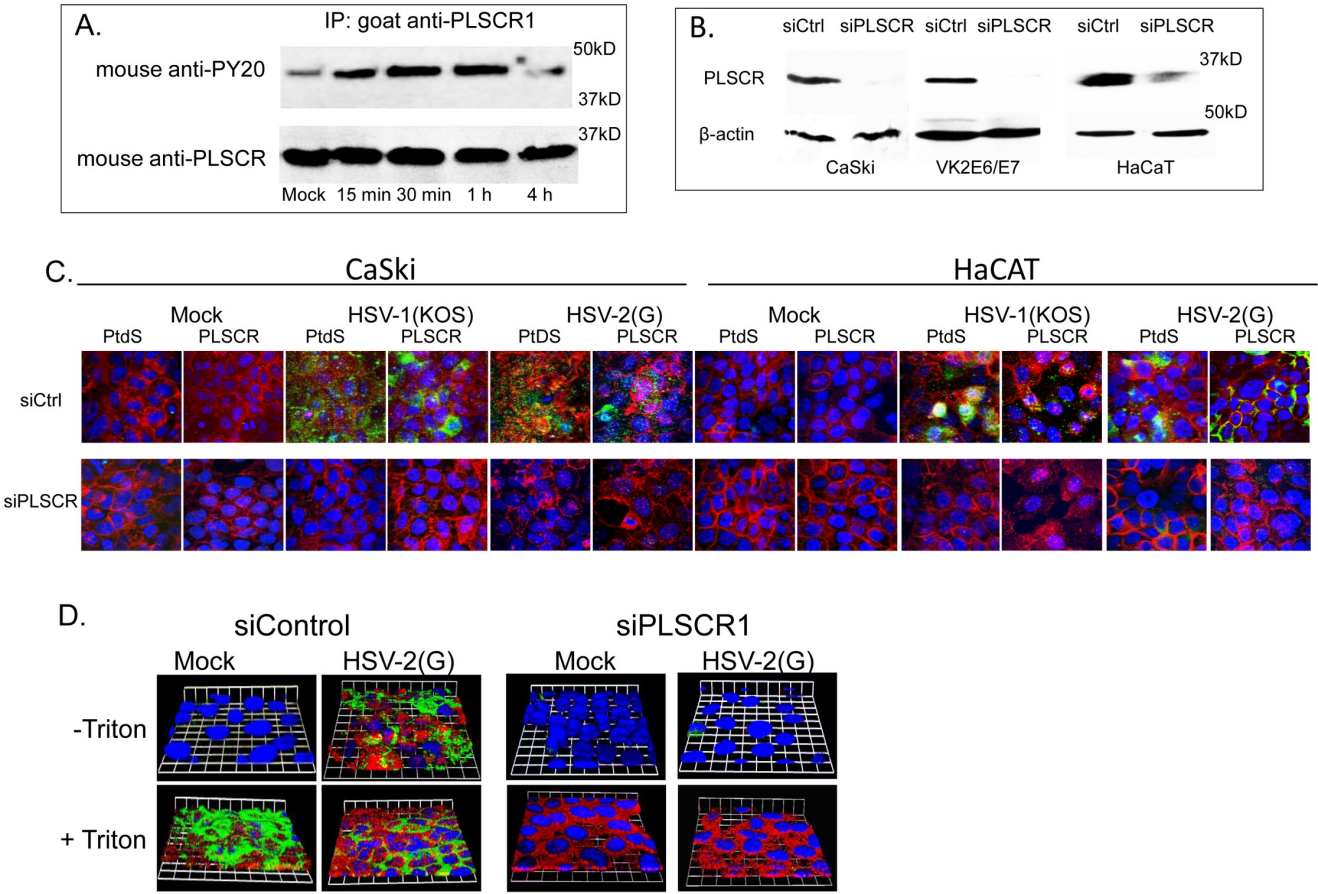
Phospholipid scramblase is required for phosphatidylserine and Akt relocalization. (A) CaSki cells were mock-infected or infected with HSV-2(G) and 15 minutes, 30 minutes, 1 hour and 4 hours post-infection, cell lysates were harvested. Lysates were incubated with a goat anti-PLSCR1 antibody and immune complexes precipitated with protein A-agarose and analyzed by Western blotting with a mouse anti-phosphotyrosine (PY20) or mouse anti-PLSCR1 mAb. The blot is representative of results obtained in 2 independent experiments. (B). CaSki, VK2E6/E7 or HaCAT cells were transfected with siRNA targeting PLSCR1 or a control siRNA (siCtrl) and protein expression was evaluated by Western blot probing for PLSCR1 (rabbit anti-PLSCR1) and β-actin (mouse monoclonal). Blot is representative of results obtained in 3 independent experiments. (C) CaSki or HaCAT cells were transfected with siControl (siCtrl) or siPLSCR1 RNA and 72 h post-transfection, plasma membranes were stained with Alexa Fluor 594-conjugated wheat germ agglutinin (red) and then synchronously infected with HSV-1(KOS), HSV-2(G), or mock-infected (4 hours at 4°C, washed, and then shifted to 37°C for 15 min and treated with low pH citrate buffer). The cells were then fixed and nuclei were stained blue with Hoechst, and phosphatidylserines (PtdS) or PLSCR1 stained green with respective primary murine and secondary Alexa 488-conjugated secondary antibodies. Images are representative of results obtained from 2–3 independent experiments. (D). CaSki cells were transfected with the indicated siRNA and then synchronously infected with HSV-2(G), fixed with or without Triton X permeabilization and stained with fluorescently-conjugated antibodies to Akt (red) and PLSCR1 (green); nuclei were stained blue with DAPI. Representative 3-D images from 3 independent experiments are shown; bars = 10μm.

We hypothesized that externalization of PtdS, and in association, Akt, may be mediated by PLSCR1. To evaluate whether HSV activates PLSCR1, CaSki cells were infected with HSV-2 or mock-infected and cell lysates were prepared at different times post-infection (pi) and incubated with an anti-PLSCR1 Ab. The immune complexes were precipitated with protein A-agarose and analyzed by immunoblotting with an anti-phosphotyrosine Ab (anti-PY20) to detect phosphorylated PLSCR1 (There is no commercially available Ab specific for phosphorylated PLSCR1). HSV induced the tyrosine phosphorylation of PLSCR1 within 15 minutes following exposure to virus and phosphorylation returned to basal levels by 4 h pi (**Fig 2A**). To test whether the HSV-induced activation of PLSCR1 was responsible for translocation of the PtdS from the inner to outer leaflet of the plasma membrane, cells were transfected with siRNA targeting PLSCR1 or control siRNA. Silencing was assessed by evaluating protein expression by Western blot analysis (**Fig 2B**) and by confocal imaging 72 h post-transfection. The cells were then synchronously infected and evaluated by confocal imaging 15 minutes post-temperature shift. PLSCR1 and PtdS were readily detected in non-permeabilized cells transfected with control siRNA in response to HSV-1 or HSV-2, but not in PLSCR1-silenced or mock-infected cells (**Fig 2C**). Moreover, silencing of PLSCR1 prevented the externalization of Akt (**Fig 2D**).

### Activation of PLSCR1 requires viral binding and gD-nectin engagement

**Fig 3:**
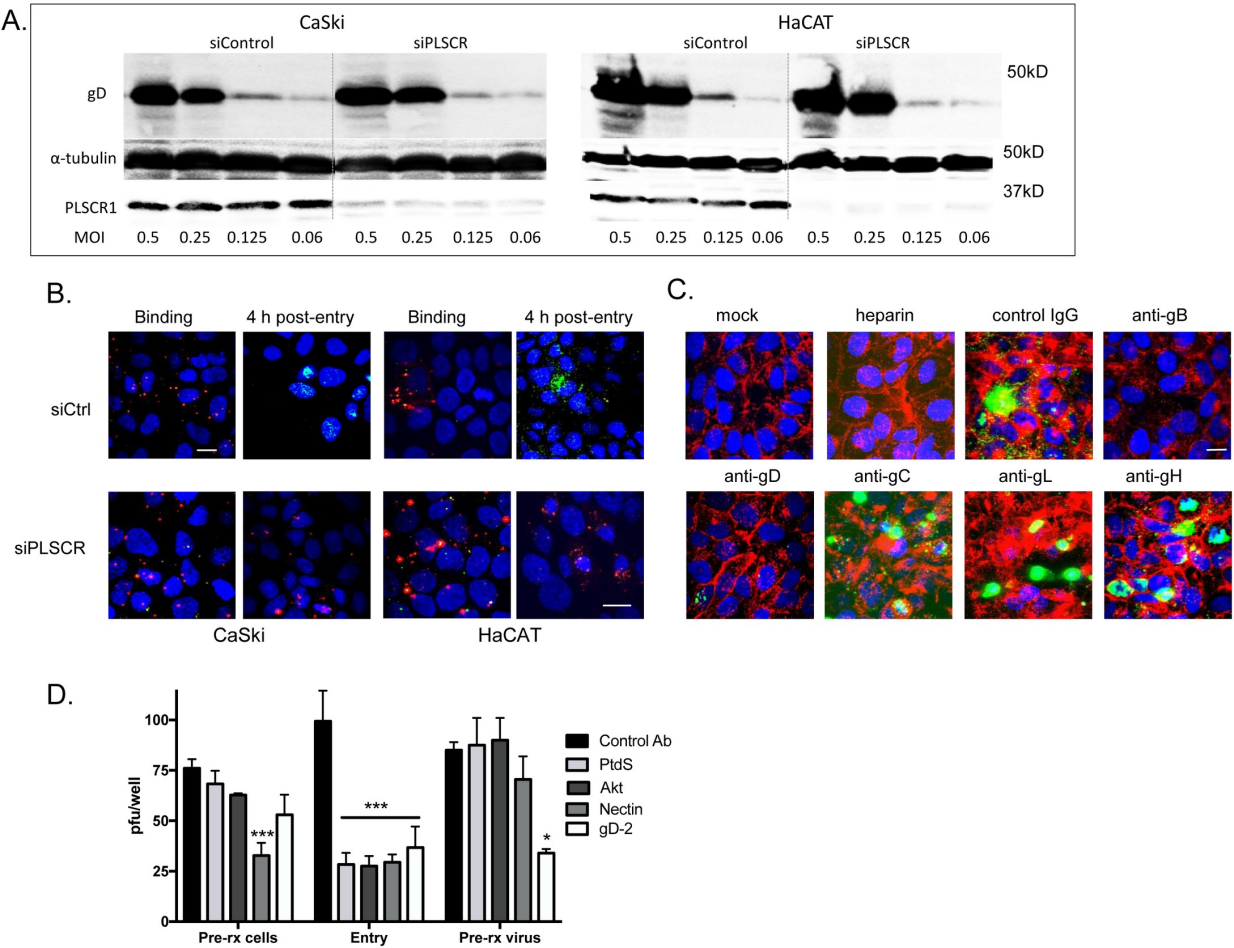
Viral binding to heparan sulfate and gD-nectin engagement precede and are required for activation of phospholipid scramblase. (A). CaSki or HaCATcells were transfected with siControl or siPLSCR1 and 72 h later were exposed to indicated multiplicities of infection (MOI) of HSV-2(G) for 4 hours at 4°C. The cells were then washed extensively and Western blots of cell lysates were probed with a mAb to gD as a marker of cell-bound virus, mAb for α-tubulin as a control for cell loading and rabbit anti-PLSCR1 as a probe for silencing. The blot is representative of results obtained in 2 independent experiments. (B). CaSki or HaCAT cells were transfected with control (Ctrl) or PLSCR1 siRNA and then infected with envelope-labeled (red) HSV-1VP26GFP (MOI 5 pfu/cell) for 4 hours at 4°C to detect binding or incubated for an additional 4 h at 37°C to detect viral entry. Results are representative of 2 independent experiments; bar = 10μm. (C). HSV-2(G) (~MOI 5 pfu/cell) was mixed with 100 μg/ml heparin or with 1:100 dilution of mAbs to HSV-2 glycoproteins gB, gD, gC, gL, or gH or control mouse IgG and then applied to CaSki cells that had been prestained with Alexa Fluor 594-conjugated wheat germ agglutinin to detect plasma membranes (red). After a 30-minute incubation, the cells were then washed, fixed and stained without permeabilization. Nuclei were stained blue with Hoechst and phosphatidylserines green with a primary murine and secondary Alexa 488-conjugated secondary antibody. Mock-infected cells are included as a control. Images are representative of results obtained from 3 independent experiments. (D). CaSki cells or HSV-2(G) (calculated to yield ~100 pfu/well) were pretreated (pre-rx) with antibodies to phosphatidylserine (PtdS), gD, Akt, nectin or control mouse IgG for 1 h and then washed 3 times (cells) or diluted 1:100 (virus-antibody mixture). Alternatively, the antibodies were added to cells at the time of viral infection for 1 h (entry). Cells were washed after the 1 h entry period, overlaid with fresh medium and plaques were quantified at 48 hours. Results are presented as pfu/well and are means+ SEM from duplicate wells in 2–3 individual experiments. The asterisks indicate p< 0.001 (***) or < 0.05 using one-way ANOVA with Dunn’s correction for multiple comparisons relative to the control.

CaSki or HaCAT cells were transfected with siPLSCR1 or siControl and exposed to serial dilutions of HSV-2(G) at 4°C. Western blots were prepared and probed for gD as a marker of bound virus as previously described [3]. Silencing of PLSCR1 had little or no impact on viral binding (**Fig 3A**). To further assess effects of PLSCR1 silencing on binding versus entry, CaSki or HaCAT cells were exposed to envelope-labeled HSV-1VP26GFP for 4 hours at 4°C or subsequently shifted to 37°C for an additional 4 h. There was no reduction in bound viral envelopes (red) in PLSCR1-silenced cells, but silencing resulted in a decrease in the percentage of GFP positive cells detected 4 h post-temperature shift (**Fig 3B**). To explore which viral envelope glycoprotein-cell interactions were required to induce PtdS externalization, HSV-2(G) was preincubated with 100 μg/ml heparin, a competitive inhibitor of viral binding, or with 1:100 dilution of mAbs to HSV-2 gB, gD, gC, gL, or gH or control mouse IgG and then applied to cells. The mAbs to gB, gD, gH and gL neutralize HSV-2 infection by ~50% at this dilution. After a 30-minute incubation, the cells were washed with low pH buffer, fixed without permeabilization and stained for PtdS. PtdS (green) were readily detected in the cells following treatment with anti-gC (gC-2 is not required for HSV-2 binding), anti-gL, anti-gH or control mouse IgG, but not when virus was incubated with anti-gB, anti-gD or heparin (**Fig 3C**). We also compared pretreating cells or concentrated virus with Abs that target PtdS, Akt, nectin, or gD and then washing the cells or diluting the virus-Ab mixture 100-fold (to a final viral inoculum that yielded ~100 plaques per well when virus was mixed with the control mouse IgG) prior to infecting cells for 1 h at 37° to allow viral entry. The cells were then washed, overlaid with medium, and plaques were counted at 48 h. Each of the Abs inhibited viral plaque formation when added during the 1 h entry period (**Fig 3D).** However, while the Abs to nectin-1 also inhibited infection when cells were pretreated, and the Abs to gD inhibited infection if the virus were pretreated, the anti-PtdS and anti-Akt Abs were only inhibitory if added during viral entry. These results are consistent with the observation that PtdS and Akt are only accessible to Abs following viral binding to heparan sulfate and engagement of nectin.

### PLSCR1 blockade inhibits HSV entry

**Fig 4:**
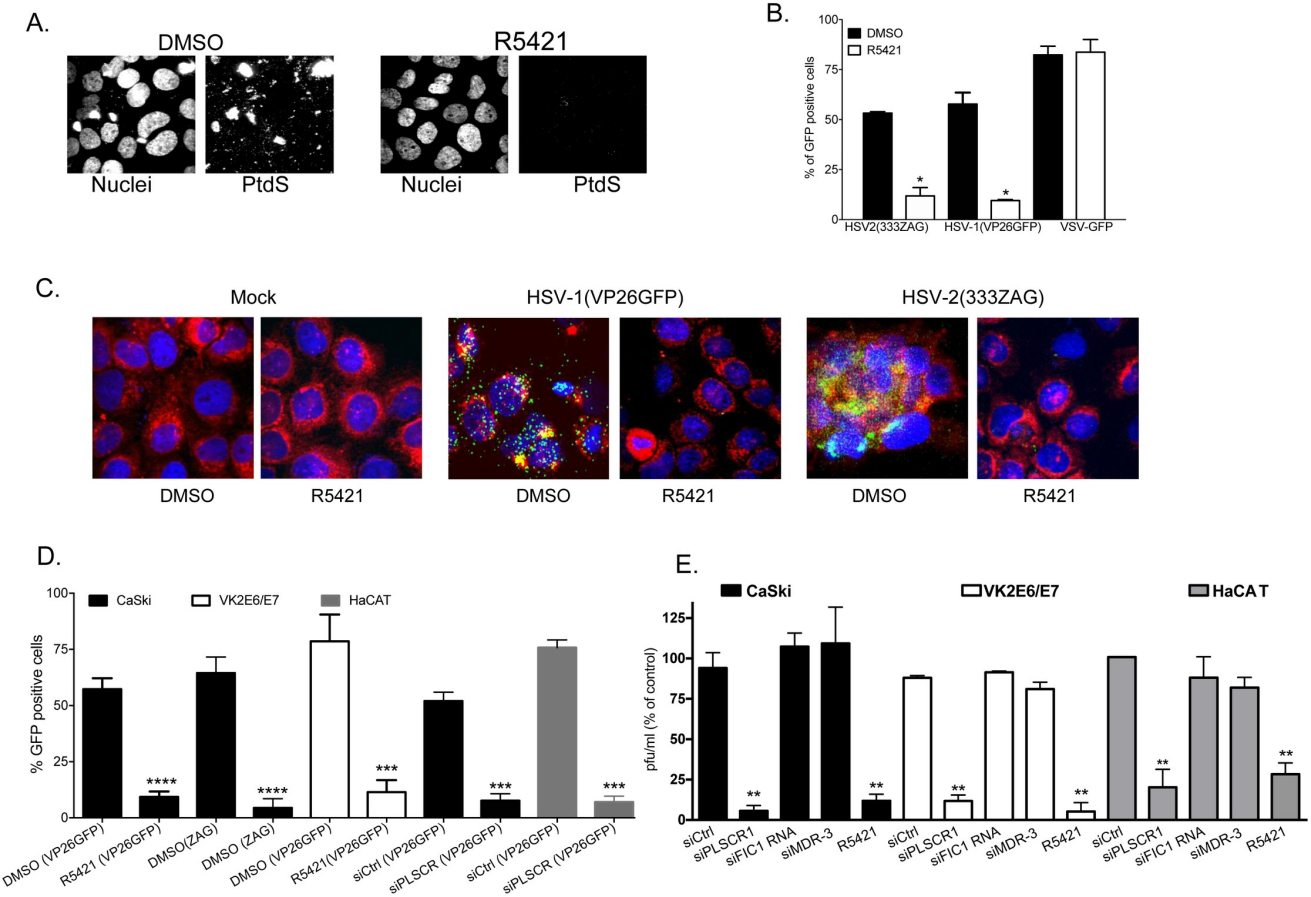
**Phospholipid scramblase blockade reduces HSV entry and viral plaque formation:** (A) CaSki cells were pretreated with 0.5% DMSO or 5 μM R5421 for 15 minutes and then infected at 37° with HSV-2(G) (10 pfu/cell) for 30 minutes. Cells were fixed and stained with fluorescently-conjugated antibodies to PtdS; nuclei were stained with DAPI. Representative images from 2 independent experiments are shown. (B) Vk2E6/E7 (HSV) or Vero (VSV) cells were pretreated with R5421 or DMSO control as in (A) and infected with HSV-2(333ZAG), HSV-1(VP26GFP) or VSV-GFP (0.1 pfu/cell). The percentage of GFP-positive cells was quantified 16 h post-infection by counting 400–500 cells in total from 4 random fields in 2 independent experiments. Results are presented as mean + SEM and the asterisks indicate p< 0.05 (student t-test compared to DMSO treated controls). (C) CaSki cells were prestained for cellular membranes (red), treated for 15 minutes with R5421 or control DMSO, and then infected with HSV-1(VP26GFP) or HSV-2(333ZAG) (~ 5 pfu/cell) for 4 h. Cells were fixed and nuclei stained blue with Hoechst. Results are representative of 2 independent experiments. (D). The percentage of GFP-positive cells detected 4 h pi is presented as mean + SEM (minimum of 200 cells were counted). The asterisks indicate significance compared to respective DMSO controls (student t-test; **** p< 0.0001; ***p< 0.001). (E). CaSki, VK2/E6E7 or HaCAT cells were transfected with siRNA targeting PLSCR1, flippase (FIC1), multidrug resistance protein-3 (MDR-3), or non-specific control (Ctrl) RNA and 72 h later were infected with HSV-2(G) (100 pfu/well). Alternatively, cells were pretreated with DMSO or R5421 15 minutes prior to infection and drug was added back to the overlay media and plaques quantified 48 h post-infection. Results are presented as percentage of pfu relative to pfu on respective control cells (siControl or DMSO) and are means from three independent experiments conducted in duplicate; asterisks indicate significance relative to controls by one-way ANOVA (** p< 0.01).

To assess whether PLSCR1 blockade inhibits viral infection, we took advantage of the pharmacological scramblase antagonist, R5421 [22]. R5421 prevented the viral induced externalization of PtdS (**Fig 4A**) and reduced HSV-2 and HSV-1, but not VSV infection (**Fig 4B**). For these studies, infection was monitored using viruses that express GFP (HSV-2(333ZAG), HSV-1(VP26GFP) and VSV-GFP) and the percentage of GFP positive cells quantified 16 h pi (n = 400–500 cells each). To more specifically assess the impact of PLSCR1 blockade on HSV entry, we again took advantage of HSV-1VP26GFP, which has GFP inserted within the VP26 capsid protein, thus providing a marker of viral entry, or HSV-2(333ZAG), which expresses GFP under the control of a CMV promoter [23]. Cells were treated with 5μM R5421 or 0.5% DMSO for 15 minutes and then infected with virus (~5 pfu/cell) for 4 h and then fixed and stained for confocal imaging. GFP expression increased within the first 4 h following inoculation with either HSV-1(VP26GFP) or HSV-2(333ZAG), but not when cells were treated with R5421 (**Fig 4C**, representative images and **Fig 4D** quantification). Note that we previously demonstrated that the increase in GFP for HSV-1(VP26GFP) reflects newly synthesized protein and was blocked by the addition of cycloheximide [15]. A similar reduction in GFP expression was observed when cells were transfected with siPLSCR1 compared to siControl and subsequently infected with HSV-1(VP26GFP) for 4 h (**Fig 4D**).

To complement the studies where GFP was used to monitor entry or infection, plaque assays were also conducted with HSV-2(G) (MOI ~0.002 pfu/cell). CaSki, VK2E6/E7 or HaCAT cells were transfected with siRNA targeting PLSCR1, multidrug resistance protein-3 (MDR-3), which encodes for an ATP-dependent floppase involved in trafficking of PtdS from the inner to outer membrane, FIC-1, or a negative control siRNA 48 h prior to infection or were treated with 5μM R541 or 0.5% DMSO just prior to infection. The efficiency of silencing ranged from 70–90% as assessed by immunoblots (S1 Fig).

Relative to siControl cells, PLSCR1 silencing reduced viral plaques by 94±6%, 89±4%, and 80±11% in the three different cell types, respectively. In contrast, siRNA targeting flippase or floppase had little impact on viral plaque formation. Similarly, R5421 reduced HSV-2 plaque formation by 88±7%, 95±6% and 71.7±7% relative to DMSO treated controls in the three cell types, respectively (**Fig 4E**).

### PLSCR1 blockade prevents Akt activation and subsequent release of Ca^2+^ stores

**Fig 5:**
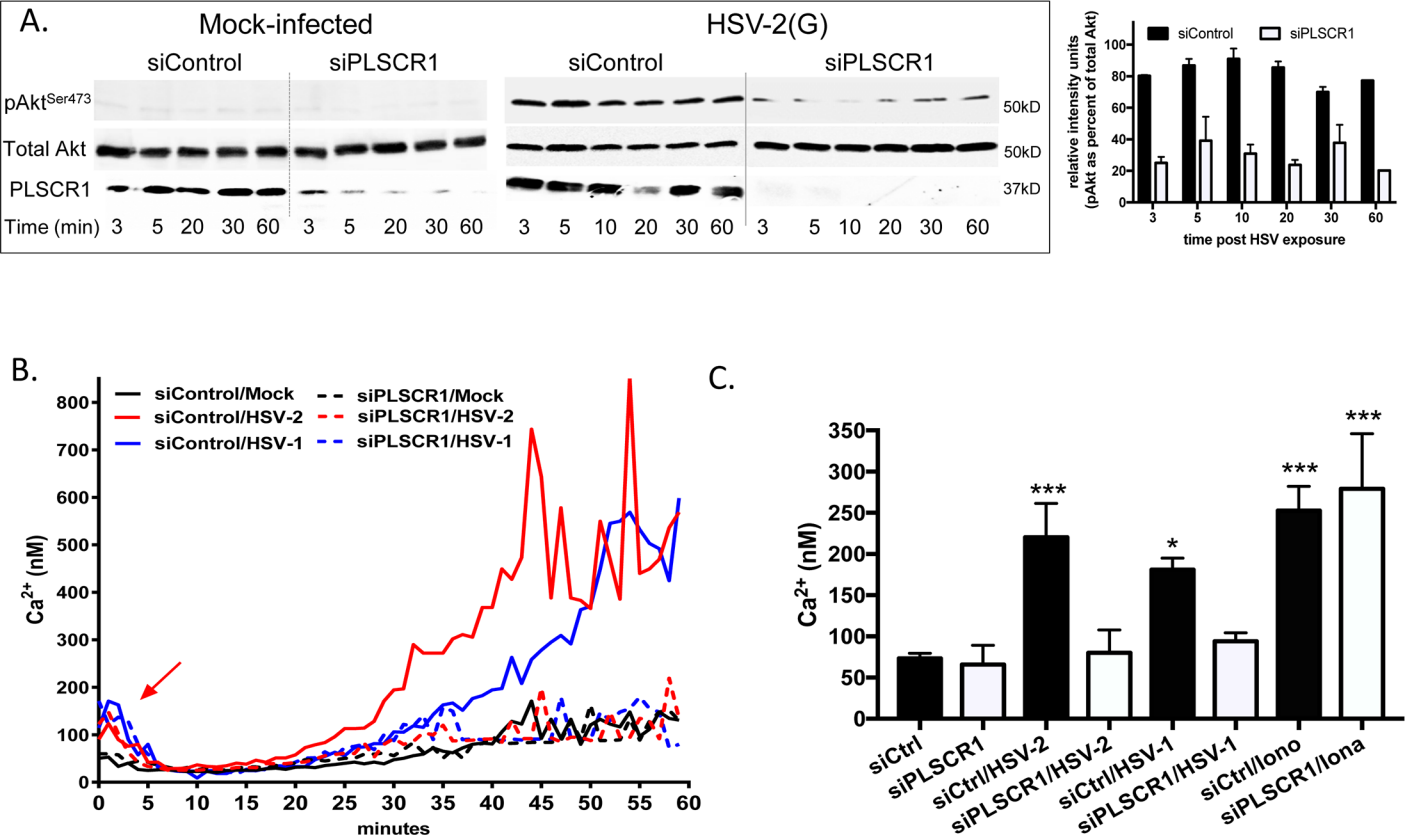
Phospholipid scramblase blockade prevents HSV-induced Akt phosphorylation, Ca^2+^ release and viral entry. (A) CaSki cells were transfected with siControl or siPLSCR1 and 72 h post-transfection were infected with purified HSV-2(G) (10 pfu/cell). Western blots of cell lysates harvested at the indicated times post-infection were probed with anti- pS^473^-Akt and then stripped and probed with anti-total Akt and anti-PLSCR1 antibodies. Representative blots from 2 independent experiments are shown. Images were scanned and pAkt as a percentage of total Akt was calculated (mean+ SEM). (B) CaSki cells were transfected with siRNA as in (A), loaded with Fura-2 and then infected with purified HSV-2(G) (5 pfu/cell), HSV-1(KOS) or mock-infected and the kinetics of calcium response monitored over 60 minutes. (C) The mean calcium released over 1 h was calculated from 4 wells in 3 independent experiments, each containing 5 x10^4^ cells. As additional controls, siRNA transfected cells were treated with ionomycin. The asterisks indicate significant differences in Ca^2+^ concentration relative to mock-infected controls (*, p<0.05, ** p<0.01, ***p< 0.001, one way ANOVA with Dunnett’s multiple comparisons test).

To evaluate whether PLSCR1 is required for Akt phosphorylation and subsequent downstream cellular responses including the release of intracellular Ca^2+^ stores, Akt phosphorylation and Ca^2+^ concentrations were monitored in siRNA transfected cells. Cells were transfected with siPLSCR1 or siControl and 72 h later were mock-infected or infected with HSV-2(G) (10 pfu/cell). Akt activation was assessed by preparing cell lysates at different times post viral exposure and assaying for phosphorylated and total Akt by Western blot and release of intracellular Ca^2+^ was monitored by fluorimetry. PLSCR1 silencing reduced Akt phosphorylation in CaSki cells in response to HSV-2(G), but had no effect on the detection of total Akt in cell lysates (**Fig 5A**). No differences in the initial (first 3–5 minutes) Ca^2+^ response (arrow) to HSV-2 or HSV-1 were observed by fluorimetry in CaSki cells transfected with siPLSCR1 compared to siControl, but the total intracellular Ca^2+^ concentration in the first hour post-viral exposure was decreased in siPLSCR1 compared to control cells (**Fig 5B and 5C**). Both serotypes triggered a significant increase in the mean intracellular Ca^2+^ concentration in siControl compared to mock-infected cells (p< 0.001 and p< 0.05, respectively), but not in siPLSCR1-transfected cells. Silencing had no discernible effect on intracellular Ca^2+^ stores as ionomycin (Ca^2+^ ionophore) induced a similar increase in the mean intracellular Ca^2+^ concentration siPLSCR1 and siControl cells. Similar results were obtained with VK2E6/E7 cells (S2 Fig).

### PLSCR1 activation is dependent on the initial Ca^2+^ response to HSV binding and gD-nectin engagement

**Fig 6:**
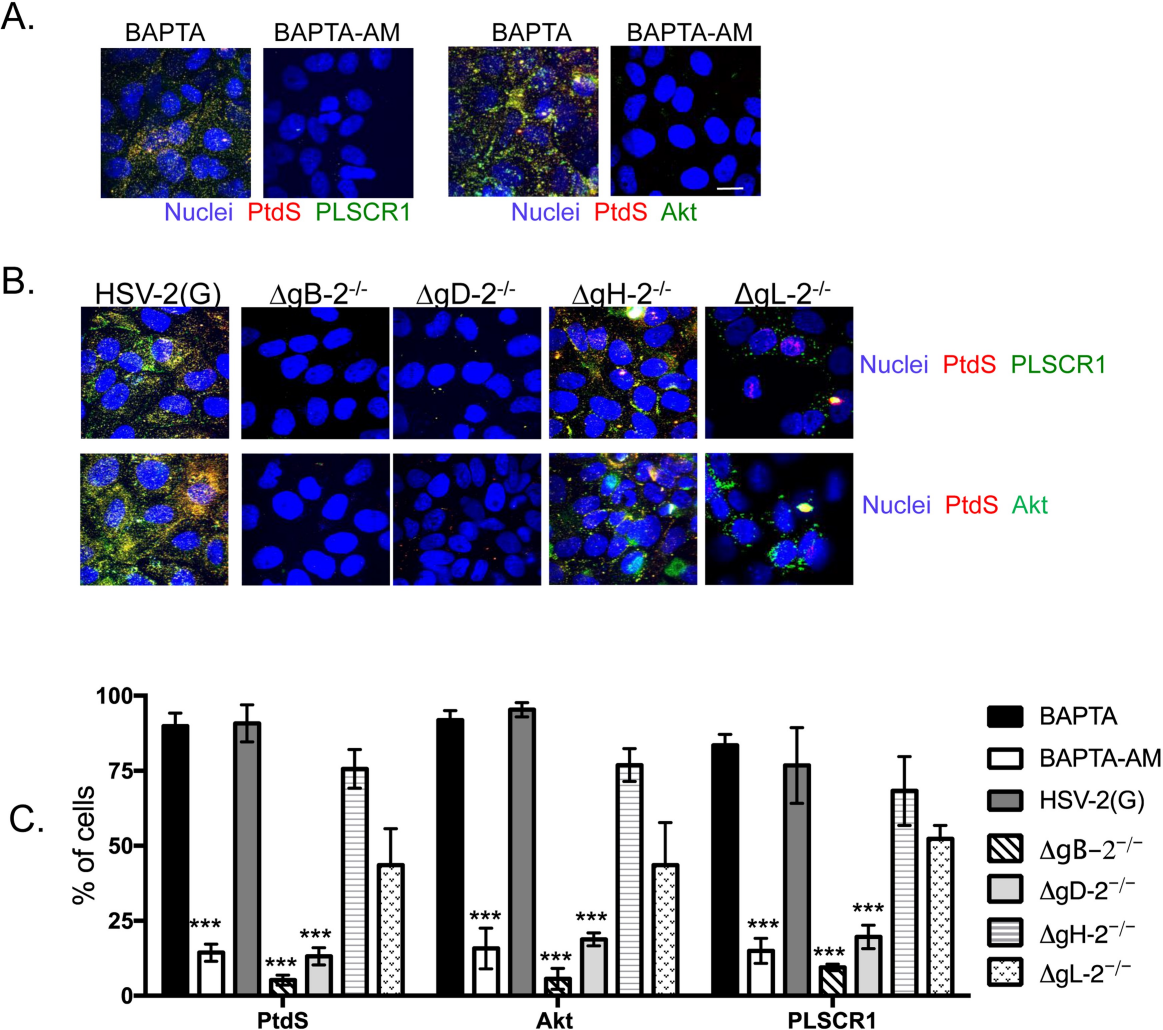
Phospholipid scramblase activation requires intracellular calcium and HSV-2 glycoprotein B and D. (A) CaSki cells were treated with 100μM BAPTA or BAPTA-AM and then infected with HSV-2(G) (10 pfu/cell) or mock-infected for 30 minutes. Cells were then fixed and stained with mAb to phosphatidylserines (PtdS) and secondary anti-mouse Alexa-555 (red), rabbit anti-PLSCR1 with Alexa-488 secondary Ab (green), or rabbit anti-Akt with Alexa-488 secondary Ab (green). Nuclei were stained blue with DAPI. Representative extended focus images from 2 independent experiments are shown (bar = 10μm). (B) CaSki cells were exposed to relatively equivalent particle numbers of HSV-2(G) or the indicated non-complemented deletion viruses (particle numbers estimated by comparing VP5 band on Western blots) for 30 minutes and fixed and stained as in panel A. Results are presentative of 4 independent experiments (2 experiments with ΔgL-2^-/-^). (C) The percentage of cells in which PtdS, Akt or PLSCR was detected by immunostaining was calculated by counting 80–100 cells in total from 4 random fields in at least 2 independent experiments (mean + SEM) and the asterisks indicate significant differences relative to HSV-2(G) (***p< 0.001, ANOVA).

We hypothesized that the early Ca^2+^ peak (Fig 5B, arrow), which we previously showed is triggered in response to HSV binding to heparan sulfate and engagement of the gD coreceptor, nectin [4,17], activates PLSCR1. To test this, cells were pretreated with 100μM of the cell permeable Ca^2+^ chelator BAPTA-AM, or the non-permeable chelator, BAPTA prior to infection, and the ability to detect PtdS, Akt, or PLSCR1 at the outer leaflet was monitored by confocal imaging 30 min following infection. BAPTA-AM, but not BAPTA, significantly reduced the number of cells with detectable PtdS, Akt, or PLSCR1 (**Fig 6A and 6C**). To further evaluate the role of the early Ca^2+^ peak in activating PLSCR1, CaSki cells were exposed to relatively equivalent particle numbers of HSV-2(G) and non-complemented viruses deleted in gB-2, gD-2, gH-2 or a newly constructed gL-2 deletion virus (HSV-2(G)ΔgL-2)) (S3 Fig). Deletion of gB-2, which plays the key role in binding of HSV-2 to heparan sulfate [3], or gD-2 (but not gH-2 or gL-2) was associated with a significant decrease in the number of non-permeabilized cells with detectable PtdS and Akt (**Fig 6B and 6C**). These findings are consistent with the observation that neutralizing Abs to gB-2 or gD-2, but not gH-2 or gL-2, prevented externalization of PtdS (Fig 3C).

### PLSCR1 interacts with glycoprotein L to promote restoration of membrane architecture

**Fig 7:**
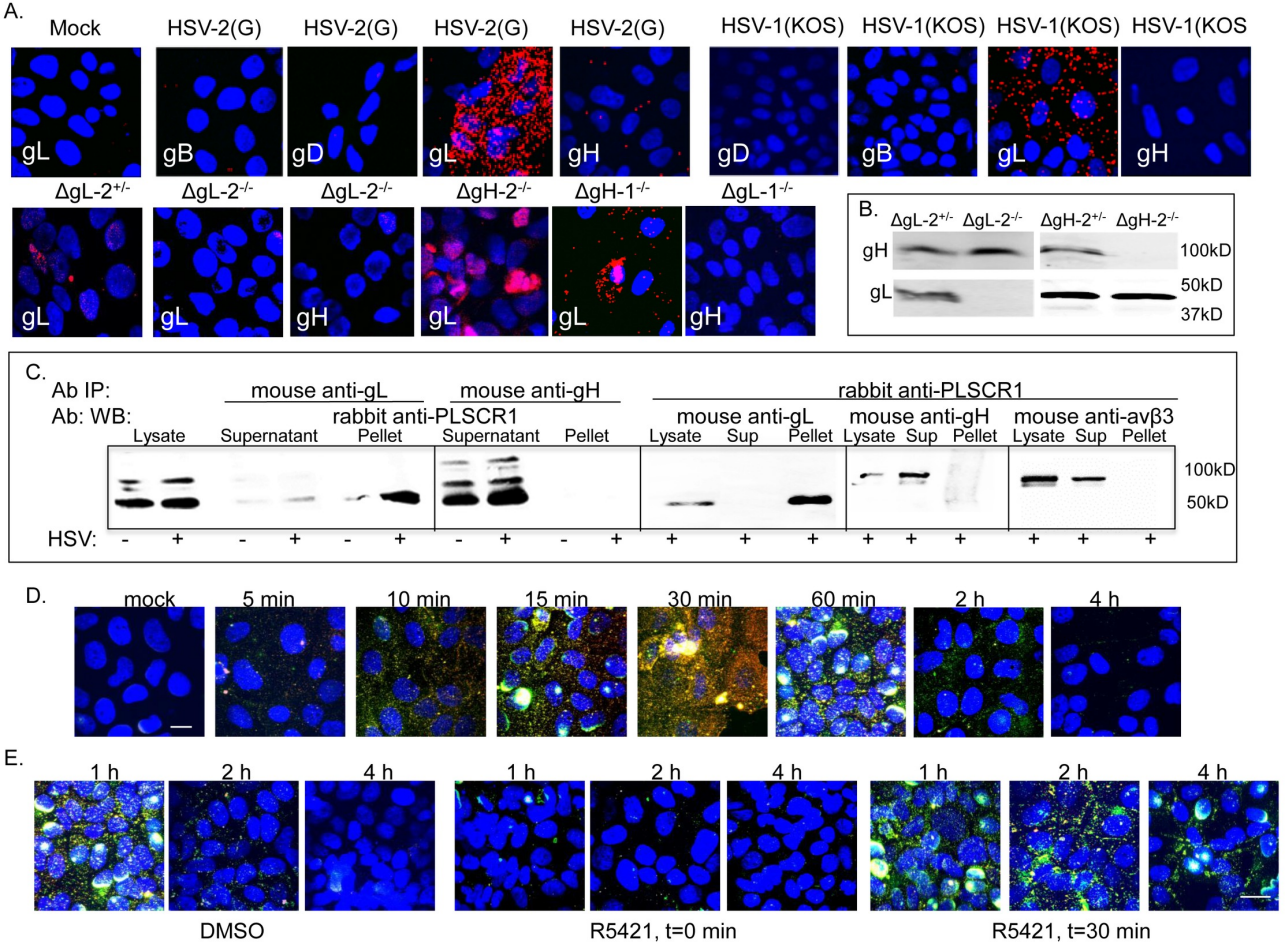
Phospholipid scramblase associates with glycoprotein L to restore membrane architecture. (A) CaSki cells were mock-infected or synchronously infected with HSV-2(G), HSV-1(KOS), complemented or non-complemented gL-2 deletion virus (ΔgL-2^+/-^ and ΔgL-2^-/-^, respectively), non-complemented gH-2 deletion (ΔgH-2^-/-^), or non-complemented gL-1 (ΔgL-1^-/-^) or gH-1 deleted viruses (ΔgH-1^-/-^) (MOI equivalent to ~ 1 pfu/cell). Fifteen minutes after the temperature shift, the cells were fixed and stained with the indicated murine HSV-serotype common mAbs and a rabbit polyclonal antibody against PLSCR1 and then probed with species-specific proximity ligation secondary antibodies. Results are representative of at least 3 independent experiments. (B). Western blots of dextran gradient-purified ΔgL or ΔgH virus isolated 24 hours after infection of 79VB4 (ΔgL-2^+/-^), F6 (ΔgH-2^+/-^) or Vero (ΔgL-2^−/−^ and ΔgH-2^-/-^) cells. Protein expression was assessed for viral glycoproteins H and L with serotype-specific mAbs. (C). CaSki cells were synchronously infected with HSV-2(G) (5 pfu/cell) (+) or mock-infected (-) and cell lysates were prepared 15 min post-temperature shift, immunoprecipitated (IP) with serotype common mouse anti-gL, mouse ant-gH or rabbit anti-PLSCR1 and equivalent volumes of supernatant, pellet or whole cell lysates analyzed by preparing Western blots (WB) and probing with rabbit anti-PLSCR1, mouse anti-gL, mouse anti-gH or mouse anti-αvβ3. Blots are representative of results obtained in 2 independent experiments. (D). CaSki cells were infected with HSV-2(G) (MOI 10 pfu/cell) and at the indicated times post-infection, cells were fixed and stained with antibodies to phosphatidylserine (red) or Akt (green); nuclei were stained blue. Mock-infected cells were included as a negative control. Representative extended focus images from two experiments are shown; scale bar 10μm. (E). CaSki cells were infected with HSV-2(G) (MOI 10 pfu/cell) for 30 minutes and then the inoculum removed, cells washed with a low pH citrate buffer and then incubated for 1, 2 or 4 h. R5421 (or DMSO) was added to the medium at the time of infection (t = 0 minutes) or immediately following citrate treatment (t = 30 minutes). Cells were stained as in Panel D. Representative images from two experiments are shown; bar = 10μm.

To determine if any of the viral envelope glycoproteins interacted with PLSCR1, we conducted proximity ligation studies. CaSki cells were synchronously infected with HSV-2(G) or HSV-1(KOS) (5 pfu/cell) and 15 minutes after the temperature shift, the cells were fixed and stained with murine serotype-common mAbs directed against gD, gB, gL or gH and with a rabbit polyclonal Ab against PLSCR1 and probed with species-specific proximity ligation secondary Abs. As a negative control for the assay, mock-infected cells were probed with mouse anti-gL and rabbit anti-PLSCR1 (**Fig 7A**). A high concentration of fluorescence (red signal) was detected only in the virally-infected cells probed with anti-gL, suggesting interactions between gL and PLSCR1 independent of gH. Because gL and gH exist, at least in part, as hetero-oligomers, additional proximity ligation studies were conducted with HSV-2(G) viruses deleted in gH or gL. An association between PLSCR1 and gL was again detected when cells were exposed to ΔgH-2 or, as a control, the phenotypically complemented ΔgL-2^+/-^ virus. In contrast, no interaction between PLSCR1 and gH was detected when cells were infected with ΔgL-2 virus; similar results were obtained with HSV-1 deletion viruses (**Fig 7A)**. Western blots of dextran purified ΔgL or ΔgH viruses grown on complementing or non-complementing cells confirmed that gH and gL are expressed independently (**Fig 7B**).

To complement the proximity ligation studies, co-immunoprecipitation studies were also performed. CaSki cells were synchronously infected with HSV-2(G) (5 pfu/cell) and cell lysates were prepared 15 min post-temperature shift and incubated with a mAb to gL or gH or conversely with rabbit anti-PLSCR1. The immune complexes were precipitated with protein A-agarose and analyzed by Western blotting with rabbit anti-PLSCR1 or, for the converse, with mAbs to gL, gH, or integrinαvβ3, which we previously showed interacts with gH downstream of Akt activation [16]. PLSCR1 was detected predominantly in the pellet following precipitation with anti-gL (but not gH), and conversely, gL (but not gH or integrinαvβ3) was detected predominantly in the pellet following precipitation with rabbit anti-PLSCR1 (**Fig 7C**).

Confocal imaging studies showed that PtdS and Akt became accessible on the outer leaflet of the plasma membrane within first 5 minutes of viral exposure, peaked at ~30 minutes but were no longer detected 2–4 h pi (**Fig 7D**), a time course that mirrors the kinetics of PLSCR1 phosphorylation (Fig 2A). The addition of R5421 at the time of infection (t = 0) prevented PtdS and Akt externalization, whereas the addition of R5421 30 min pi resulted in persistence of PtdS and Akt at the outer leaflet for up to 4 h pi (**Fig 7E**), indicating a role for PLSCR1 in the bidirectional movement of PtdS and Akt during HSV entry.

**Fig 8:**
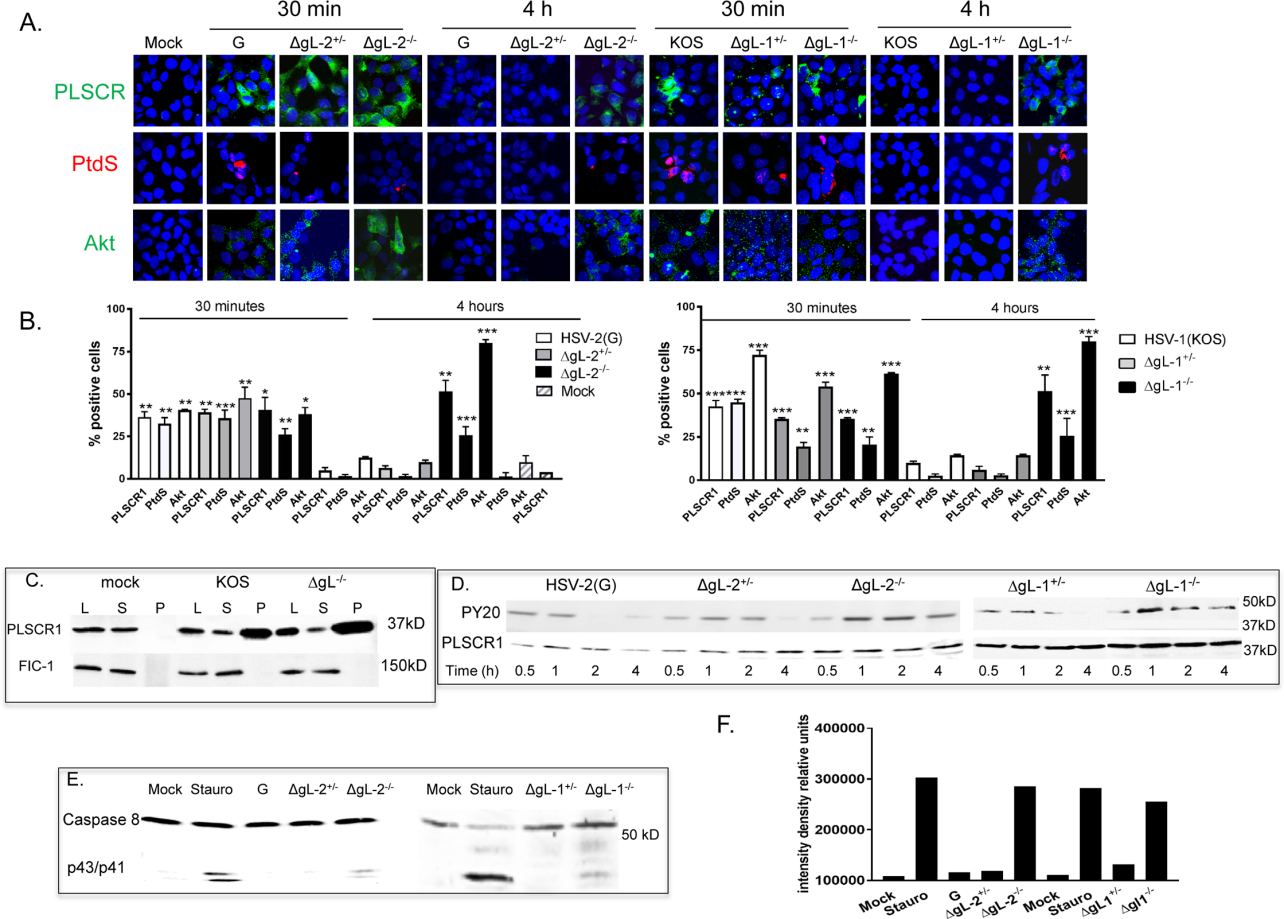
Phosphatidylserines persist at the outer leaflet when cells are exposed to viruses deleted in glycoprotein L. (A). CaSki cells were mock-infected or infected with HSV-2(G), complemented or non-complemented ΔgL-2 (ΔgL-2^+/-^ or ΔgL-2^-/-^), KOS, and complemented or non-complemented ΔgL-1 (ΔgL-1^+/-^ or ΔgL-1^-/-^) viruses (MOI ~ 1 pfu/cell) and then fixed and stained 30 min or 4 h pi for PLSCR1 (green) (rabbit polyclonal anti-PLSCR1 and secondary Alexa 488 Ab), PtdS (red) (annexin-V Alexa 555) or Akt (green) (rabbit polyclonal and secondary Alexa 488 Abs). Representative images from 2–4 experiments are shown). (B). The percentages of cells with detectable PLSCR1, PtdS and Akt were determined by counting 80–100 cells in two independent experiments; results are means + SEM (* p< 0.05; ** p< 0.01, *** p< 0.001, ANOVA compared to mock-infected cells). (C). CaSki cells were mock-infected or synchronously infected with equivalent particles numbers of HSV-1(KOS) or HSV-1ΔgL^-/-^ and harvested 15 min after the temperature shift. Cell surface proteins were biotinylated and precipitated with streptavidin magnetic beads and equivalent volumes of supernatant (S) and pellet (P) were analyzed by immunoblotting with Ab to scramblase; controls include whole cell lysates (L). Blots were stripped and probed with rabbit-anti-FIC-1 Ab. Results are representative of 2–3 independent experiments. (D). Cells were infected with indicated viruses (1 pfu/cell) and at the indicated times pi, cellular lysates prepared, incubated with rabbit anti-PLSCR1 Ab overnight, and immune complexes precipitated with protein G-agarose and analyzed by Western blotting with a mouse anti-phosphotyrosine mAb (PY20) or mouse anti-PLSCR1 Ab. The blot is representative of results obtained in 2 independent experiments. (E). CaSki cells were mock-infected or exposed to each of the indicated viruses (MOI ~ 1 pfu/cell) or 10 μM staurosporine and 4 h pi, cellular lysates were prepared and analyzed by immunoblotting for caspase-8. A representative blot from two independent experiments is shown and (F) is the relative intensity density of the scanned p43/p41 band in relative units.

Deletion of gL had no effect on the activation of PLSCR1 as evidenced by the increase in PLSCR, PtdS and Akt detected by confocal imaging in non-permeabilized cells 30 minutes post-viral exposure compared to mock-infected cells (**Fig 8A and 8B)** or by biotinylation of membrane proteins and immunoblotting for PLSCR1 (or as a control, FIC-1) (**Fig 8C**). However, while these cellular proteins were no longer detected by confocal microscopy 4 h pi following exposure to HSV-2(G) or to the complemented ΔgL viruses, they persisted following exposure to the non-complemented ΔgL^-/-^ viruses (**Fig 8A and 8B**). These findings paralleled the kinetics of phosphorylated PLSCR1, which persisted 4 h post-exposure to the noncomplemented, but not complemented ΔgL viruses (**Fig 8D**). Persistence of externalized PtdS was also associated with an increase in apoptosis as measured by caspase-8 cleavage (p43/p41) (**Fig 8E and 8F**).

### Ionomycin also triggers activation of PLSCR and Akt

**Fig 9:**
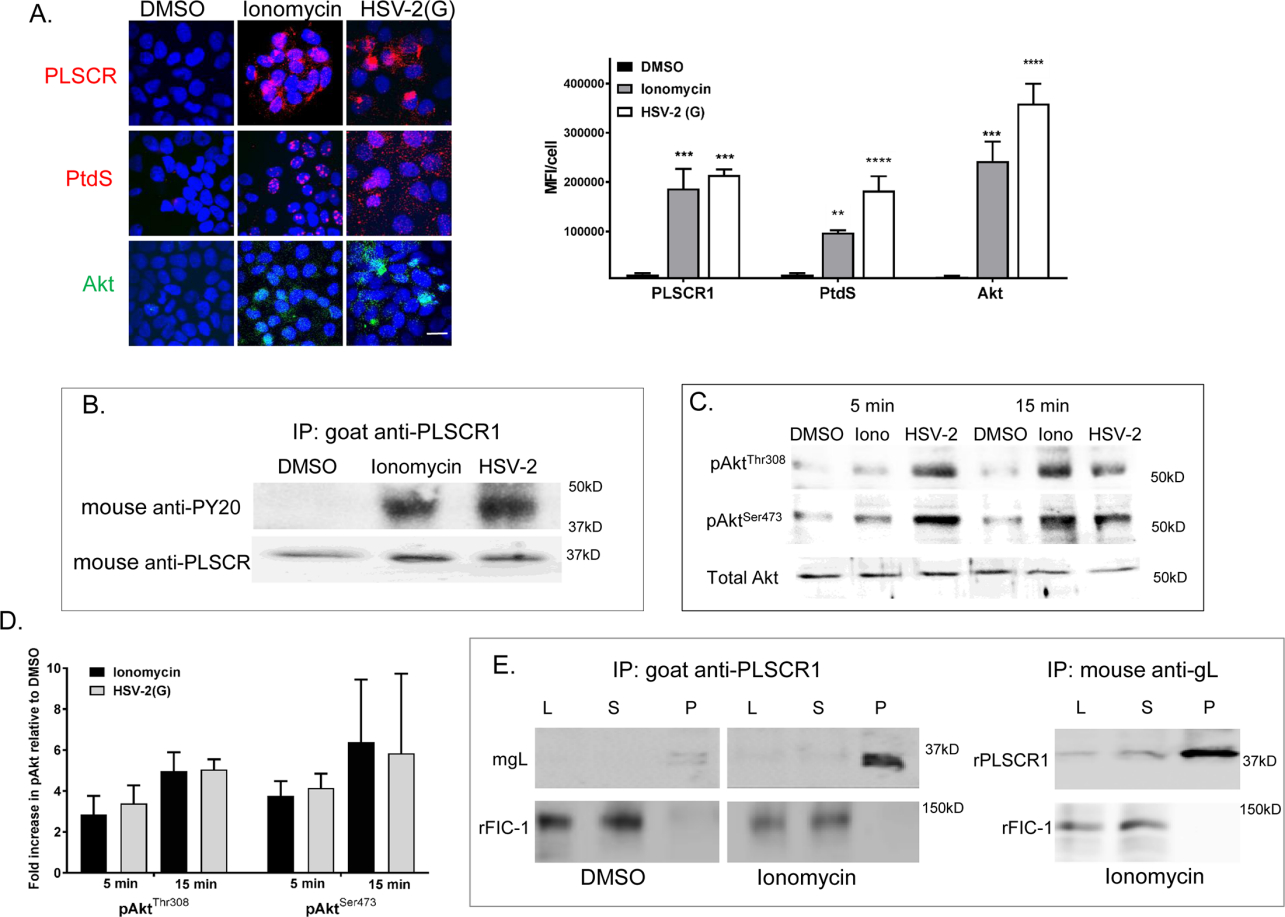
Ionomycin activates phospholipid scramblase leading to externalization of phosphatidylserines and Akt. (A). CaSki cells were treated with ionomycin (1μM), HSV-2(G) (MOI 5 pfu/cell) or DMSO (0.1%) for 15 minutes and then fixed and stained with antibodies to PLSCR1(polyclonal rabbit and Alexa 555, red), PtdS (monoclonal antibody and Alexa555, red), or Akt (polyclonal rabbit and secondary Alexa488, green) as indicated. Nuclei were stained blue with DAPI. Images are representative of results obtained from 2–3 independent experiments; bar = 10μm. Five fields were scanned and mean fluorescence intensity (MFI) per cell calculated using ImageJ software (NIH); results are mean +SEM and the asterisks indicate significant differences by ANOVA compared to DMSO control treated cells. (B). CaSki cells were treated as in A for 15 minutes and then cell lysates were harvested, incubated with a goat anti-PLSCR1 antibody and immune complexes precipitated with protein G-agarose and analyzed by Western blotting with a mouse anti-phosphotyrosine mAb (PY20) or mouse anti-PLSCR1 Ab. The blot is representative of results obtained in 2 independent experiments. (C). CaSki cells were harvested 5 or 15 minutes after exposure to DMSO, 1μM ionomycin (Iono) or HSV-2(G) (5 pfu/cell). Cell surface proteins were biotinylated and precipitated with streptavidin magnetic beads and the pellet analyzed by immunoblotting with rabbit anti-pAktThr^308^ or mouse anti-pAktS^473^ Abs. In parallel, cellular lysates were analyzed by immunoblotting for total cellular Akt (rabbit polyclonal Ab). Blots representative of 2 independent experiments are shown. (D). The blots were scanned and fold increase in pAktThr^308^ and anti-pAktS^473^ relative to DMSO treated cells is depicted (mean+ SEM). (E). HaCAT cells were treated with 1μM ionomycin or DMSO (0.1%) for 10 minutes and then incubated with soluble gL for 30 minutes at 37°C, transferred to ice, and immunoprecipitated (IP) with goat anti-PLSCR1 (left) or with mouse-anti-gL (right). Equivalent volumes of whole cell lysate, supernatant, or pellet (L, S, P) were analyzed by preparing Western blots (WB) and probing with mouse anti-gL, rabbit anti-PLSCR1 or rabbit anti-FIC-1 as a control. The blots are representative of results obtained in 2 independent experiments.

To determine whether ionomycin, a Ca^2+^ ionophore, has a similar impact on PLSCR1 and Akt as was observed with virus, we exposed cells to HSV-2(G), ionomycin or DMSO for 5 or 15 minutes [24]. Both ionomycin and HSV-2(G) activated PLSCR1 resulting in the detection of PLSCR1, PtdS, and Akt in non-permeabilized cells by microscopy (**Fig 9A)** and an increase in phosphorylated PLSCR1 (**Fig 9B**) and phosphorylated Akt (both at the threonine 308 and serine 473 sites) (**Fig 9C and 9D**) by immunoblotting. Notably, when cells were incubated with recombinant gL for 30 minutes and immune complexes precipitated with an anti-PLSCR1 Ab, gL was detected in the pellet primarily in the ionomycin-treated but not the control (DMSO)-treated cells (**Fig 9E**). Similar results were obtained when reciprocal co-immunoprecipitation studies were performed and complexes were immunoprecipitated with anti-gL and immunoblots probed with anti-PLSCR. These findings support an interaction between gL and activated PLSCR1 independent of other viral glycoproteins.

## Discussion

**Fig 10:**
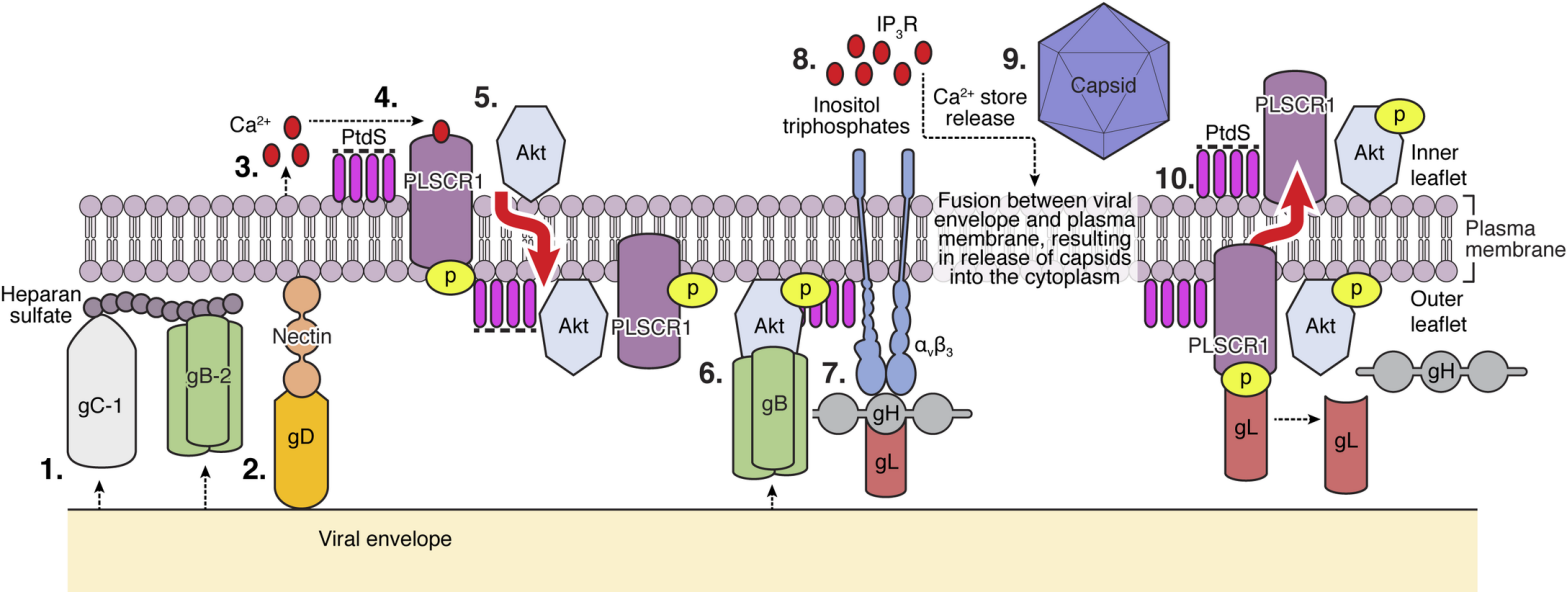
Model of phospholipid scramblase activation during HSV entry. Binding of HSV-1 glycoprotein C or HSV-2 glycoprotein B (Step 1) and engagement of nectin-1 by glycoprotein D (Step 2) trigger the release of a small amount of calcium near the plasma membrane (Step 3) that is sufficient to activate (phosphorylate) phospholipid scramblase (Step 4). This results in flipping of phosphatidylserines (PtdS) from the inner to the outer leaflet of the plasma membrane, which results in externalization of Akt and possibly other inner plasma membrane- associated proteins (Step 5). Akt interacts with gB, which may promote a conformational change in Akt that facilitates its phosphorylation at threonine 308 and serine 473 by yet to be determined kinases (Step 6). Integrinαvβ3 binds to gH (Step 7), and activated Akt and integrin transfer signals to the cell cytoplasm that lead to the generation of inositol triphosphates and release of inositol-triphosphate receptor (IP3R)-regulated Ca^2+^ stores (Step 8) culminating in fusion of the viral envelope and plasma membrane and entry of viral capsids (Step 9). PtdS flip back to the inner leaflet of the plasma membrane to restore normal membrane asymmetry in a process that may occur in response to viral entry and may be regulated by gL binding to PLSCR1 and subsequent shift to primarily dephosphorylated PLSCR1. (Step 10).

These studies identify a previously undescribed and novel role for PLSCR1 in HSV entry and provide a key missing link in understanding how Akt, which has been presumed to shuttle between the cytoplasm and the inner leaflet of the plasma membrane, gains access to viral envelope glycoproteins at the outer leaflet [17]. Our findings demonstrate that HSV-1 and HSV-2 activate PLSCR1 and suggest the model illustrated in **Fig 10**.

In response to viral gC (HSV-1) and/or gB (HSV-2) binding to heparan sulfate and engagement of the primary co-receptor, nectin-1, by gD (both serotypes), a small amount of Ca^2+^ is released intracellularly, which is sufficient to activate PLSCR1. PLSCR1 flips PtdS from the inner to outer leaflet of the plasma membrane, which results in externalization of Akt and possibly other inner leaflet proteins. Glycoprotein B binds to externalized Akt, which may promote a conformational change to expose Akt phosphorylation sites, similar to what happens when Akt interacts with 3’-phosphoinositides at the inner plasma membrane [25]. Akt becomes phosphorylated by yet to be determined kinases at serine 473 and threonine 308 and activated Akt may act as a catalytic kinase, possibly via outside-in signaling, although the downstream targets of Akt’s kinase activity require future study. Interference with PLSCR1 (chelation of intracellular Ca^2+^, siRNA, or R5421) or Akt (siRNA, miltefosine [26] and other Akt kinase inhibitors) prevents downstream signaling events associated with viral entry including interactions between integrin αvβ3 and gH, generation of inositol triphosphates and release of IP3R-dependent intracellular Ca^2+^ stores [4,14,16,17].

Externalization of Akt had not been previously described [17], but may be a generalized response to PLSCR1 activation, as phosphorylated Akt was also detected on the outside when cells were treated with ionomycin, a Ca^2+^ ionophore. Prolonged PtdS exposure on the outer leaflet of the plasma membrane is an apoptotic signal [21]. HSV prevents this by flipping PtdS back within 2–4 h following viral exposure in a process that is dependent on PLSCR1 and gL. PtdS persist on the outside if R5421 is added to the cultures 30-minutes post-infection or if the cells are infected with HSV variants deleted in gL. The major role of gL in HSV entry has been presumed to be that of a chaperone protein, enabling gH to be transported from the ER to the plasma membrane where it is incorporated into the viral envelope as a gH-gL heterodimer [27]. However more recent work indicates that gL becomes dissociated from gH during viral entry and may play a regulatory role [28]. Our findings indicate that at least part of this “regulatory” role involves PLSCR1. HSV may initially “lock” PLSCR1 in a conformation that retains PtdS and Akt at the outer leaflet to allow Akt activation but, subsequently, in a process that involves gL, HSV “unlocks” PLSCR1 to allow PtdS to flip back thereby preventing apoptosis. Our studies also suggest that the conformation and activation state of PLSCR1 are linked to its tyrosine phosphorylation. PLSCR1 is tyrosine phosphorylated and readily detected by immunostaining with a mAb that recognizes an N-terminal epitope in non-permeablized cells shortly after exposure to HSV-1 or HSV-2. However, 2–4 h pi, PLSCR1 becomes inactivated and is detected only in permeabilized cells and primarily in its non-phosphorylated form. In contrast, phosphorylated PLSCR1 persists and remains detectable in non-permeabilized cells following exposure to HSV-1 or HSV-2 gL deletion viruses. We speculate that interactions between gL and PLSCR1 may alter the conformation and thus the phosphorylation state of PLSCR1.

Equine herpesvirus (EHV)-1 also activates PLSCR1, but this was observed downstream of the interactions between gH and integrin α4β1 and was dependent on the release of IP3R-regulated intracellular Ca^2+^ stores [29]. In contrast, HSV-1 and HSV-2 activate PLSCR1 upstream and independent of the interactions between gH and integrins. Possibly this reflects difference in the cellular response to gD co-receptors. Both EHV and HSV bind to heparan sulfate moieties [5,7,30], but EHV-1 gD interacts with MHC class 1 molecules [31,32], whereas HSV gD interacts with nectin-1 on most cell types. HSV binding and gD-nectin interactions trigger the release of intracellular Ca^2+^ near the plasma membrane, a response that is necessary and sufficient to activate PLSCR1. Blockade of Ca^2+^ release with the cell-permeable chelator, BAPTA-AM, inhibiting heparan sulfate binding with soluble heparin, or gD-nectin-1 interactions by adding antibodies to gD or nectin to the cultures, prevents PLSCR1 activation.

Several viruses incorporate PtdS into their envelope and then exploit this to interact with PtdS cellular receptors such as Human T-cell Immunoglobulin and Mucin-domain containing proteins (TIMs) to promote subsequent entry or egress [33]. For example, Ebola virus VP40 interacts with PtdS during viral egress causing the relocalization of PtdS from the inner to outer leaflet of the plasma membrane [34]. Whether this involves PLSCR1 or other scramblases has not been addressed. The PtdS on the viral envelope subsequently bind TIM-1 or TIM-4 to mediate viral entry into target cells. Similar mechanisms are employed by flaviviruses such as dengue [33]. In contrast, the results of the current studies including the silencing of cellular PLSCR1 suggest that the source of the PtdS is not the HSV envelope but rather flipping of cellular PtdS from the inner to the outer plasma membrane in response to the increase in intracellular Ca^2+^ observed when virus binds to heparan sulfate and engages nectin. The addition of anti-PtdS antibodies during viral entry reduced viral infection suggesting a direct or indirect role for PtdS in this process that warrants future investigation. Possibly, PtdS promote Akt-gB binding analogous to the observation that PtdS promote binding of Akt to phosphatidylinositol-3,4,5-triphosphate (PIP3) and subsequent conformational changes needed for Akt phosphorylation and activation [35].

The key role of PLSCR1 and Akt in HSV-1 and HSV-2 entry into several different human cell types and their localization to the outer leaflet of the plasma membrane where they interact with HSV glycoproteins L and B, respectively, represents an unprecedented opportunity for targeting these molecules to develop new antivirals. Potentially membrane-impermeable Akt or PLSCR1 inhibitors could be developed that would specifically block HSV entry without adversely impacting normal cellular signaling pathways.

## Materials and methods

### Cells and viruses

CaSki [human cervical epithelial cancer cell line; American Type Culture Collection (ATTC) CRL_-_1550, Manassas, VA, USA], VK2/E6E7 [human papilloma virus E6/E7 transformed vaginal epithelial cells ATTC CRL-2616], and Vero [monkey kidney epithelial cell line; ATCC CCL-81] cells were passaged in DMEM supplemented with 10% fetal bovine serum (FBS); HaCAT cells [human keratinocyte spontaneously immortalized cell line; ATTC CLS 300493] were propagated in DMEM supplemented with 10% FBS. The viral isolates HSV-2(G), [HSV-1(KOS), HSV-1(KVP26GFP), which contains a green fluorescent protein (GFP)-VP26 fusion protein [23], HSV-2(333ZAG), which expresses GFP under the control of a cytomegalovirus (CMV) promoter inserted in an intergenic region between UL3 and UL4 (gift from P. Spear, Northwestern University), and VSV-GFP, which expresses EGFP under the control of the VSV polymerase (gift from K. Chandran, Albert Einstein College of Medicine) were propagated and titered on Vero cells. An HSV-2 (G) virus deleted in gD (ΔgD-2) [17] was propagated on HSV-1 gD-expressing VD60 cells [36], an HSV-2(G) virus deleted in gB (ΔgB-2) was propagated on HSV-2 gB expressing VgB2 cells [3], an HSV-2(G) virus deleted in gH-2 (Cheshenko N., 2014) was propagated on HSV-1 gH expressing F6 cells [37]. An HSV-1(KOS) virus deleted in gL (ΔgL-1) was propagated on gL-1 expressing 79VB4 cells [14] and an HSV-1(KOS) virus deleted in gH (ΔgH-1) (gift from P. Spear) was propagated on F6 cells [34]. Complemented viruses were passaged once on Vero cells to obtain glycoprotein-negative virions. The complemented and non-complemented viruses were purified on dextran gradients, titered on complementing and non-complementing cells, and relative particle numbers determined by probing Western blots with an antibody to the capsid protein VP5 [4]. A corresponding aliquot from the weight balance gradient used during centrifugation was included as a control in indicated experiments. In select studies viral envelopes were labeled by incubating with a lipophilic tracer DiL (Invitrogen) for 10 min at room temperature before purification on sucrose gradients [4].

### Construction of a gL-2 deletion virus

79VB4 cells were co-transfected with HSV-2(G) DNA and DNA from an engineered plasmid (designated pEF6/5UL1HygroGFP) that contains the gL-2 gene (UL1) disrupted at the BmgBI and HpaI sites (R0628, R0105, New England BioLabs) by the hygromycin enhanced green fluorescent protein (EGFP) fusion protein under the control of the immediate early promoter of human cytomegalovirus (HCMV) constructed from pHygGFP (Clontech) (The plasmid encoding HSV-2(G) gL-2, designated pEF6/5 UL1 was a gift from Torin Weisbrod and William Jacobs).This dual function marker vector allows for drug selection with the ability to identify positive transfectants using GFP as a fluorescent reporter. Cotransfections were performed using the Effectene Transfection reagent (301425, Qiagen). Recombinants were selected on 79VB4 cells for ability to grow in the presence of 200 *μ*g/ml hygromycin and expression of EGFP (10687010, Thermo Fisher). Selected recombinants were plaque-purified three times and subsequently analyzed for absence of gL and GFP protein expression by Western blot analysis and for deletion of nucleotides 439–688 by PCR using viral DNA isolated 24 h pi of Vero cells (S1 Fig and Fig 7A).

### Antibodies and chemical reagents

Primary antibodies and dilutions were as follows: mouse anti-PtdS mAb, 1:200 (05–719, Millipore, Upstate Biotechnology, Lake Placid, NY); mouse anti-PLSCR1 mAb, 1:200 (ab24923, Abcam Cambridge, MA), rabbit anti-PLSCR1, 1:500 (NBP1-322588, NOVUS Biologicals, Littleton, CO); goat anti-PLSCR1 (sc-27779, Santa Cruz Biotechnology); mouse anti-phosphotyrosine mAb (4G10; 05-1050X, Millipore,); mouse anti-nectin 1 (HA265, Virusys Taneytown, MD); mouse anti-MDR-3 mAb,1:200 (sc-58221, Santa Cruz Biotechnology); rabbit anti-FIC1, 1:500 (sc-134967, Santa Cruz Biotechnology), mouse anti-β-actin mAb, 1:5000 (A-5441, Sigma-Aldrich); mouse anti-phospho-Akt (Ser-473) mAb, 1:500 (4051, Cell Signaling Technology, Danvers, MA); rabbit anti-phospho-Akt (Thr-308) mAb, 1:500 (9275, Cell Signaling Technology); rabbit anti-Akt123, 1:1000 (sc-8312, Santa Cruz Biotechnology); mouse anti- integrinαvβ3 mAb, 1:200 (sc- 7312, Santa Cruz Biotechnology); mouse anti-VP5 mAb (serotype common), 1:200 (sc-56989, Santa Cruz Biotechnology); mouse anti-gB-2 mAb, 1:500 (sc-69799, Santa Cruz Biotechnology); mouse anti-gB mAb (serotype common), 1:100 (HA056, Virusys); mouse anti-gD-2 mAb, 1:1000 (sc-56988, Santa Cruz Biotechnology); mouse anti-gD (serotype common) mAb, 1:100 (HA025-100, Virusys); mouse anti-gL-1 mAb 1:200 (H1A259-100, Virusys); mouse anti-gH-1 mAb, 1:200 (H1A258-100, Virusys); mouse anti-gL-2 mAb,1:100 (HA262-100, Virusys); mouse anti-gH-2 mAb,1:100 (H2A261-100, Virusys); mouse anti-gC-2 mAb,1:100 (H2A260-100, Virusys); rabbit anti-goat IgG, 1:500 (STAR AR122, Bio-Rad), mouse IgG, 1:100 (sc-2025, Santa Cruz Biotechnology); mouse anti-α tubulin, 1:500 (62204, Thermo Fisher Scientific), and rabbit anti-GFP, 1:250 (ab32146, Abcam). Annexin V Alexa Fluor 555 was purchased from Thermo Fisher Scientific (A 35108). The secondary antibodies for Western blots were horseradish peroxidase-conjugated goat anti-mouse (170–5047, Bio-Rad, Hercules, CA), goat anti-rabbit (170–5046, Bio-Rad), and donkey anti-goat 1:1000 (sc-2020, Santa Cruz). The secondary antibodies for confocal microscopy were goat anti-mouse Alexa Fluor 350 (A-11045, Invitrogen Molecular Probes), goat anti-mouse Alexa Fluor 555 (A-21147, Thermo Fisher), goat anti mouse Alexa Fluor 488 (A11001, Thermo Fisher) and goat anti-rabbit Alexa Fluor 488 (A-11078, Thermo Fisher) or Alexa Fluor 555 (A-21428, Thermo Fisher). All secondary antibodies were diluted 1:1000. R5421 was purchased from Sigma Aldrich (ANV00195), ionomycin (I24222) and staurosporine (PHZ1271) from Invitrogen Molecular Probes, and heparin sodium salt (H-3393) from Sigma Aldrich. Recombinant gL was purchased from MyBiosorce (MBS1463300).

### Small interfering RNA (siRNA) transfections

Cells were transfected with 10 nM of the indicated siRNA sequences in 12-well plates using the HiPerFect Transfection Reagent (1029975, Qiagen). Human PLSCR1 siRNA (sc-44028), FIC1 siRNA (sc-62317), and MDR-3 siRNA (sc-37015I) were purchased from Santa Cruz Biotechnology (Santa Cruz, CA, USA) and a control siRNA (Cat# AM4636) was purchased from Applied Biosystems (Applied Biosystems, Foster City, CA). Cells were analyzed for protein expression by preparing Western blots of cell lysates 72 h post-transfection.

### Confocal microscopy

Cells were grown on glass coverslips in 12- or 24-well plates, and, where indicated, were transfected with siRNA and 72 h post-transfection were infected with virus or mock-infected (media or dextran control). To label plasma membranes and nuclei, the cells were stained for 10 min with red-fluorescent Alexa Fluor 594 wheat germ agglutinin and blue-fluorescent Hoechst 33342 Image-IT LIVE Plasma Membrane and Nuclear Labeling Kit or with green-fluorescent Alexa Fluor 488 wheat germ agglutinin (I34406, W11261, Invitrogen Molecular Probes, Carlsbad, CA, USA) before infection. Cellular membranes were stained with cell trace BODIPY ester (I34407, Invitrogen Molecular Probes). In select experiments, cells were infected at 4°C for 1 or 4 h to allow virus to bind, washed with PBS three times to remove unbound virus, and then shifted to 37°C for 15 minutes to allow entry. Virus that had not yet penetrated was inactivated with a low pH citrate buffer [14]. Cells were fixed with 4% paraformaldehyde solution (Electron Microscopy, Hatfield, PA, USA) with or without permeabilization with 0.1% of Triton-X for 3 min. Nuclei were counterstained with DAPI (D1306, Invitrogen). PtdS were stained with annexin-V or anti-PtdS Abs and flippase was stained with anti-FIC-1 Abs. Images were acquired by laser confocal microscope ZeissLive/DuoScan equipped with oil immersion objectives 63×1.4 and 100×1.4. Images were captured in an optical slice of ~0.37 μm with appropriate filters; Alexa Fluor 488 and GFP were excited using the 488-nm line of a krypton/argon laser and viewed with a 505- to 530-nm band pass μm; AlexaFluor 360 was excited with 405-nm diode laser and collected with 420 to 475 nm filter; AlexaFluor 555 was excited using 561-nm helium/neon laser and collected with a 575 to 655 filter. All images were captured using the multitrack mode of the microscope to decrease cross talk of fluorescent signals (Zeiss LSM); 3D and extended focus images were generated using Volocity 5.3 software (Improvision, Lexington, MA). The number of PLSCR1, Akt or PtdS positive cells was quantified using Cell Counter ImageJ software (NIH). Monitoring of GFP expression in live cells was performed using Nikon microscope equipped with objective 4.

### Flow cytometry

HaCAT cells (2x10^6^ cells/sample) were harvested using Accutase solution (A6964, Sigma Life Science), washed and exposed to purified HSV-2(G) or ΔgD-2 virus (purified from Vero cells [ΔgD^-/-^]) at a MOI 1 or 10 pfu/cell based on Vero titer for HSV-2(G) or a relatively equivalent number of viral particles based on VP5 expression on Western blots for ΔgD-2) or control buffer for 20 minutes in suspension. The cells were then placed on ice, stained with Live-or-Dye Fixable Viability Staining Kit (32002, Biotuium, Inc. Fremont, CA) and then stained for PtdS using PE-Annexin V (556421, BD Pharmingen). The cells were fixed with 4% paraformaldehyde solution. Flow cytometry was performed on a Becton Dickinson LSR II analyzer and data analyzed using the FlowJo v9.3.1 software (TreeStar, Inc., Ashland, OR). Thirty thousand live events were acquired per sample.

### Western blots to assess Akt phosphorylation

Cells were serum-starved for 24 h and then exposed to live virus (or media supplemented with 0.5% of DMSO or dextran as a control for purified viruses) and at different times post-viral exposure, the cells were harvested and lysed in buffer containing 20 mM Tris pH 7.5, 50 mM NaCl, 1% NP-40, 0.05% DOC, supplemented with fresh protease and phosphatase inhibitors (118735, Roche Diagnostics, and P0044, P5726, Sigma Aldrich, respectively). Proteins were separated by SDS-PAGE and transferred to membranes for immunoblotting with the indicated antibodies. Membranes were stripped between antibodies by incubating in Restore Western Blot Stripping Buffer (21059, Thermo Scientific) for 10 min at room temperature and washing 3 times in TBS-Tween. Blots were visualized, scanned and the band intensities were analyzed using ChemiDoc imaging system equipped with GelDoc2000 software (RRID:SCR_014210, Bio Rad). Western blots were quantified using ImageJ software (NIH).

### Biotinylation of cell surface proteins

CaSki cells were exposed to HSV-2 (MOI 10 PFU/cell) or mock infected at 4°C for 4 h. At different times following transfer of the cells to 37°C, cells were washed 4 times with ice-cold PBS and biotinylated with sulfo-NHS-SS-Biotin (F20650; Invitrogen Molecular Probes) for 1 hour at 4°C. After 3 washes with PBS supplemented with 1% BSA, cells were harvested, solubilized in PBS containing a proteinase inhibitor cocktail, precipitated with streptavidin magnetic beads (Dynabeads M-280 Streptavidin; Life Technologies, Gaithersburg, MD, USA) and analyzed by immunoblotting.

### Viral plaque assays

Cells were exposed to the indicated dilutions of virus for 1 h at 37°C, washed once with a low pH citrate buffer and then three times with phosphate buffered saline (PBS, pH 7.4), and overlaid with medium 199 containing 1% pooled human IgG (I2511, Sigma Aldrich). Plaques were counted by immunoassay using an anti–human IgG antibody peroxidase conjugate (PA1-86064, Thermo Fisher) [6]. In select experiments, cells were pretreated for 15 minutes with R5421 or 0.5% DMSO in DMEM as a control.

### Calcium kinetic measurements

CaSki or VK2E6/E7 cells (5x10^4^) were seeded in 96 well black plates with clear bottoms (3340, CellBIND surface, Corning Inc., NY) and incubated with 25 μM Fura-2 AM diluted in PBS (F1221, Invitrogen Molecular Probes) for 60 min at 37°C, rinsed with PBS thrice, placed on ice and then exposed to cold purified HSV-2 (MOI~5 pfu/cell) or control buffer (PBS). In select experiments, cells were pretreated with 5μM R5421 prior to infection. Additional controls included cells exposed to 1μM of ionomycin. The cells were then transferred to SpectraMaxMF^e^ temperature-regulated chamber at 37°C (Molecular Devices Ca) without washing; photometric data for intracellular Ca^2+^ concentration [Ca^2+^] were generated by exciting cells at 340 and 380nm and measuring emission at 510 nm every minute for one hour using SoftMaxPro. 5.4 software (Molecular Devices). An intracellular calibration was performed with each experiment by determining the fluorescence ratio (340:380) in the presence of Ca-free 10mM K_2_EGTA buffer (R_min_) and 10mM CaEGTA buffer containing 10μM ionomycin (R_max_) (C-3008, Calcium Calibration Buffer Kit #1, Invitrogen Molecular Probes). The mean [Ca^2+^] was determined from four wells according to the manufacturer’s recommendations using the following equation: [Ca^2+^] = K_d_ Q (R-R_min_)/(R _max_-R), where R represents the fluorescence intensity ratio F_λ1_/F_λ2_; λ1(340 nm) and λ2 (380 nm) are the fluorescence detection wavelengths for ion-bound and ion-free indicators; K_d_ is the Ca^2+^ dissociation constant and equals 0.14 μM (Fura and Indo Ratiometric Calcium Indicators, Invitrogen Molecular Probes); and Q is the ratio of F_min_ to F_max_ at λ2 (380nm).

### Co-immunoprecipitation assays

Cells were serum-starved for 24 h prior to being exposed to virus (equivalent to 5 pfu/cell) by for 4 h at 4°C, washed 3x with cold PBS, and then placed in a water bath at 37°C for 15 min to allow viral entry and then immediately placed back on ice. Alternatively, serum-starved cells were treated with ionomycin (or DMSO control) for 10 min and then exposed to soluble gL for 30 min before being placed back on ice. The cells were then lysed by sonication in RIPA buffer (Thermo Scientific) supplemented with complete protease inhibitors (Roche Diagnostics). The lysates were incubated overnight at 4°C with mAb to gL or gH, control mouse IgG, or rabbit-anti-PLSCR1 and then immune complexes were isolated following a 4-h incubation with protein A agarose beads (Thermo Scientific) or protein G Plus agarose beads (sc-500778, Santa Cruz Biotechnology). The precipitated complexes (pellet), supernatants or an aliquot of the cell lysate were analyzed by Western blot with rabbit anti-scramblase polyclonal Abs or mAbs to gL, gH, gB, or gD, nectin or integrinαvβ3.

Because there is no Ab for phosphorylated scramblase, co-immunoprecipitation was also used to identify phosphorylated scramblase-1. Cells were serum starved overnight, infected with virus (5 pfu/cell) or serum-free medium (mock-infection) and cell lysates were prepared at different times and incubated with goat-anti-PLSCR1 Ab (sc-27779, Santa Cruz Biotechnology). The immune complexes were precipitated with protein A-agarose and analyzed by Western blotting with mouse anti-phosphotyrosine mAb.

### Proximity ligation assay

Cells were infected synchronously as described for co-immunoprecipitation studies and then, after 15 minutes, transferred to 37°C, fixed with cold 4% paraformaldehyde and then incubated with the indicated primary Abs (1:100, 3% BSA in PBS) for 1 h at room temperature. PLA probes were anti-Mouse MINUS (Duolink in Situ, DUO092004, Sigma Aldrich) and anti-Rabbit PLUS (Duolink, in Situ, OLK-92002-0100, DUO092002, Sigma Aldrich). Blocking, hybridization, ligation, amplification and detection steps were performed following the manufacturer’s protocol (Detection Reagents Orange, DUO092007, mounting medium with DAPI (Duolink, 82040–0005; Sigma-Aldrich). Fluorescence signals were detected using Zeiss Live DuoScan confocal microscope fitted with a 63 ×1.4 oil objective. Z-sections were captured in an optical slice of 0.44μm. Extended focus images were generated using the Volocity 5.3. confocal software (Improvision, Lexington, MA).

### Apoptosis assay

CaSki cells (1x10^6^ cells /well in 6-well plates) were infected with purified complemented or non-complemented ΔgL virus (MOI equivalent to 1 pfu/cell). After 1 h of incubation, the cells were treated with low pH citrate buffer, washed three times with media and overlaid with serum-free media. Controls included cells treated with 10 μM staurosporine (positive control) or an aliquot from the weight balance dextran gradient (negative control). Cellular lysates were prepared after 4 h of incubation. Proteins were separated by SDS-PAGE and transferred to membranes for immunoblotting with an anti-caspase 8 mAb (1: 200, NB500-208, NOVUS Biologicals, Littleton, CO).

### Statistical analyses

Analyses were performed using GraphPad Prism version 7.0 software (GraphPad Software Inc. San Diego, CA). A *P* value of 0.05 was considered statistically significant. Results were compared using unpaired Student's *t* tests or ANOVA as indicated.

## Supporting information

S1 Fig Efficiency of siRNA silencing. Vk2E6/E7 cells were transfected with siRNA targeting flippase (FIC1) (A), multidrug resistance protein-3 (MDR-3 (B) or non-specific control (Ctrl) siRNA and 72 hours later, protein expression was monitored by preparing Western blots and probing for flippase, MDR-3 or, as a control, β-actin. The blot is representative of results obtained in two independent experiments.
(TIF)

S2 Fig Phospholipid scramblase silencing reduces intracellular Ca^2+^ release in Vk2E6/E7 cells. Vk2E6/E7 cells were transfected with siRNA targeting phospholipid scramblase 1 (siPLSCR1) or a control siRNA and 72 hours post-transfection, loaded with Fura-2 and then infected with purified HSV-2(G) (5 pfu/cell) or mock-infected. The calcium responses were monitored over 60 minutes.
(TIF)

S3 Fig Characterization of HSV-2 ΔgL virus. (A). DNA was purified from uninfected Vero cells (mock, control) or Vero cells infected for 24 h with HSV-2(G) or the plaque purified gL deletion virus that had been passaged on complementing 79VB4 cells (ΔgL-2^+/-^) (MOI 1 pfu/cell based on titer on Vero or 79VB3 cells, respectively). The presence of the first 362 nucleotides of UL1 (left panel) and deletion of nucleotides 439–688 (right panel) were assessed by PCR using the indicated primer sets. (B). CaSki cells were infected with HSV-1(G) or the complemented gL deletion virus (ΔgL-2^+/-^) at a MOI of 1 pfu/cell and cell lysates were harvested 8 and 16 h pi and analyzed by performing Western blots with polyclonal anti-GFP Ab or monoclonal anti-gL-2 Ab. Results are representative of 2 independent experiments.
(TIF)
