# Supplementary figures and images for: Herpes simplex viruses activate phospholipid scramblase to redistribute phosphatidylserines and Akt to the outer leaflet of the plasma membrane and promote viral entry
Source: PLoS Pathog. 2018 Jan 2;14(1):e1006766. doi: 10.1371/journal.ppat.1006766 (PMC5766253; doi:10.1371/journal.ppat.1006766)

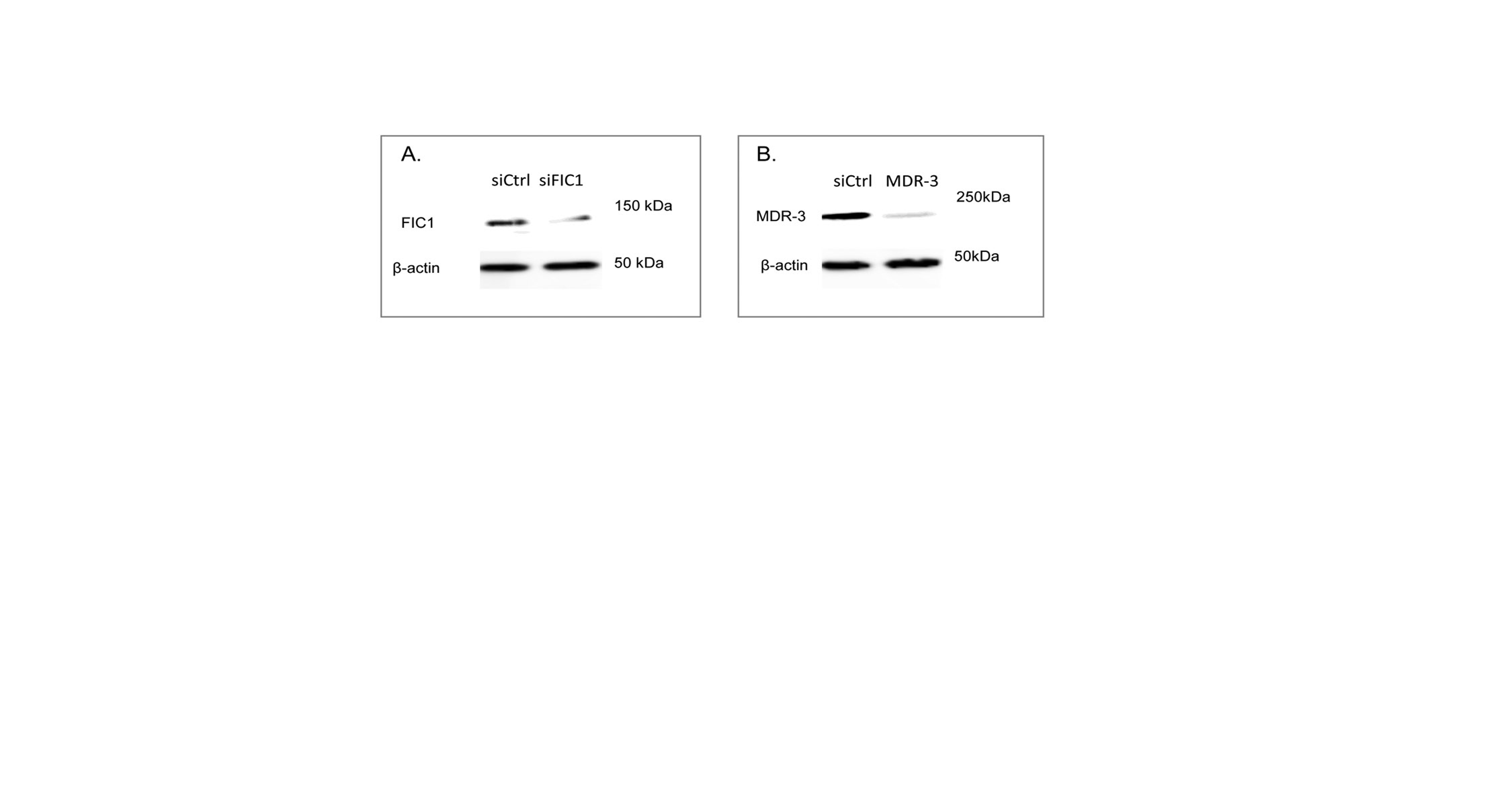

Supplement: S1 Fig — Vk2E6/E7 cells were transfected with siRNA targeting flippase (FIC1) (A), multidrug resistance protein-3 (MDR-3 (B) or non-specific control (Ctrl) siRNA and 72 hours later, protein expression was monitored by preparing Western blots and probing for flippase, MDR-3 or, as a control, β-actin. The blot is representative of results obtained in two independent experiments. (TIF) [file ppat.1006766.s001.tif]

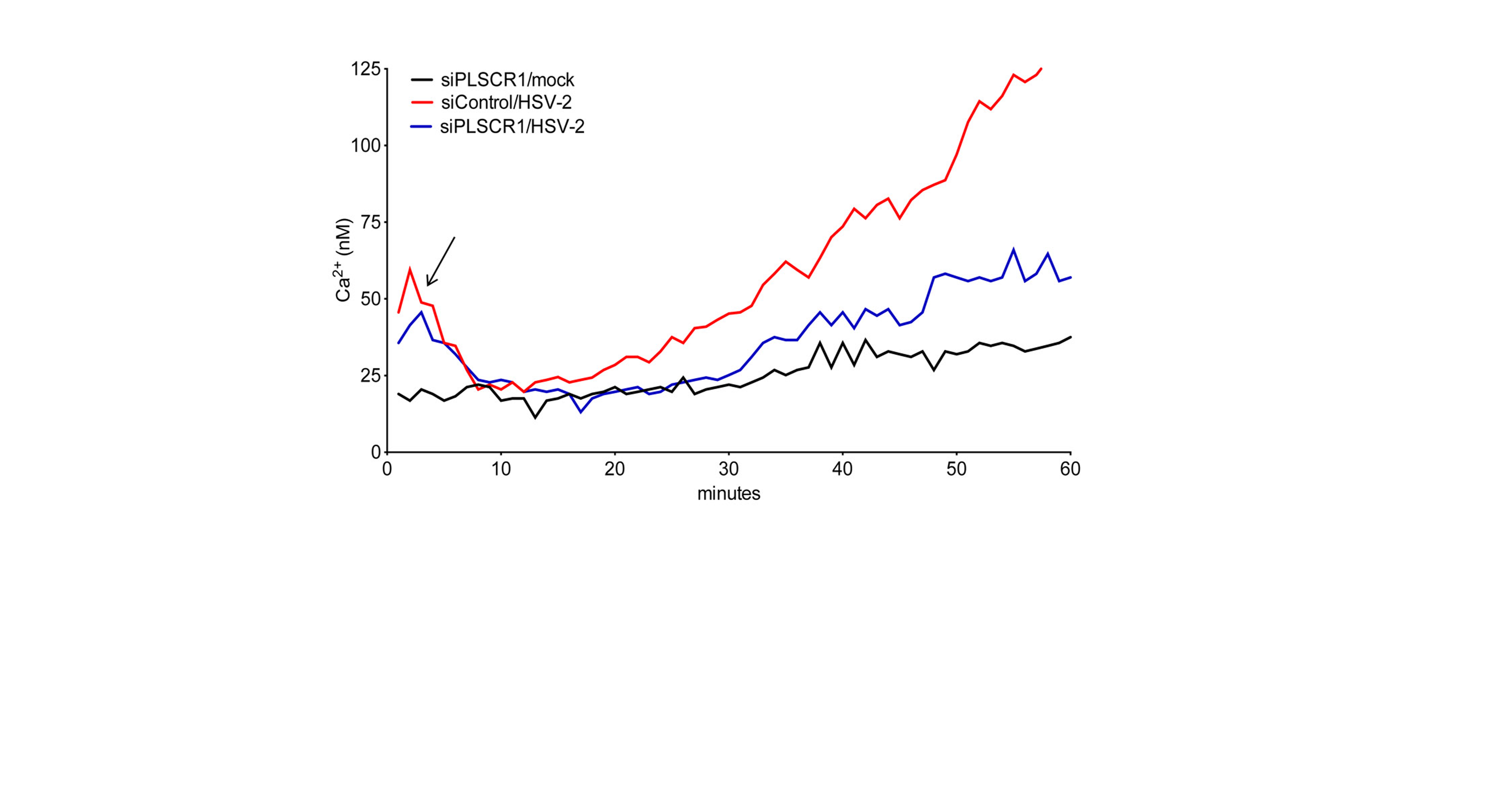

Supplement: S2 Fig — Vk2E6/E7 cells were transfected with siRNA targeting phospholipid scramblase 1 (siPLSCR1) or a control siRNA and 72 hours post-transfection, loaded with Fura-2 and then infected with purified HSV-2(G) (5 pfu/cell) or mock-infected. The calcium responses were monitored over 60 minutes. (TIF) [file ppat.1006766.s002.tif]

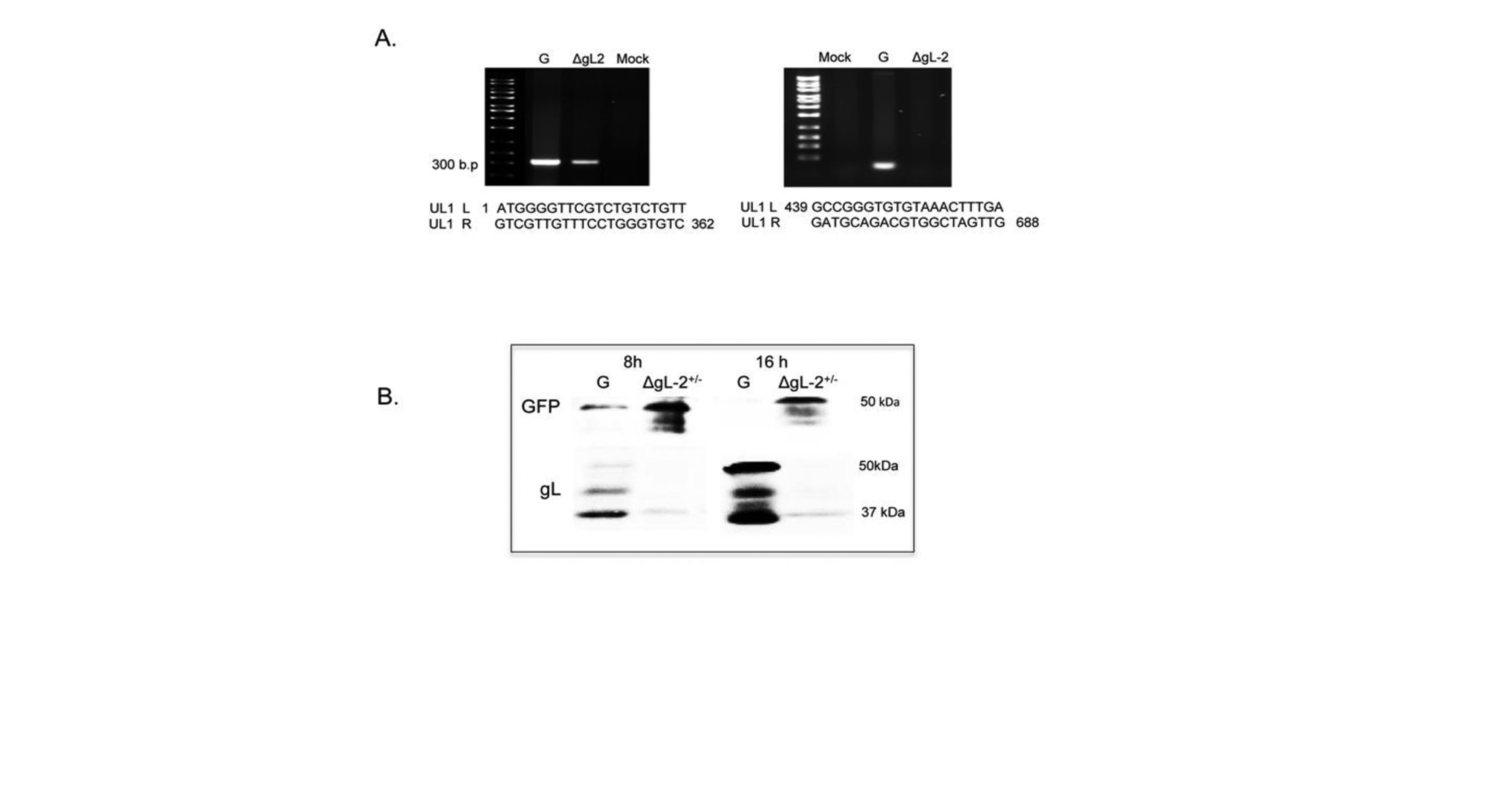

Supplement: S3 Fig — (A). DNA was purified from uninfected Vero cells (mock, control) or Vero cells infected for 24 h with HSV-2(G) or the plaque purified gL deletion virus that had been passaged on complementing 79VB4 cells (ΔgL-2+/-) (MOI 1 pfu/cell based on titer on Vero or 79VB3 cells, respectively). The presence of the first 362 nucleotides of UL1 (left panel) and deletion of nucleotides 439–688 (right panel) were assessed by PCR using the indicated primer sets. (B). CaSki cells were infected with HSV-1(G) or the complemented gL deletion virus (ΔgL-2+/-) at a MOI of 1 pfu/cell and cell lysates were harvested 8 and 16 h pi and analyzed by performing Western blots with polyclonal anti-GFP Ab or monoclonal anti-gL-2 Ab. Results are representative of 2 independent experiments. (TIF) [file ppat.1006766.s003.tif]
